# Network Pharmacology-Based Strategy to Investigate the Pharmacological Mechanisms of *Ginkgo biloba* Extract for Aging

**DOI:** 10.1155/2020/8508491

**Published:** 2020-07-27

**Authors:** Yanfei Liu, Yue Liu, Wantong Zhang, Mingyue Sun, Weiliang Weng, Rui Gao

**Affiliations:** ^1^Graduate School of Beijing University of Chinese Medicine, Beijing 100029, China; ^2^Institute of Clinical Pharmacology of Xiyuan Hospital, China Academy of Chinese Medical Sciences, Beijing 100091, China; ^3^Cardiovascular Diseases Center, Xiyuan Hospital of China Academy of Chinese Medical Sciences, Beijing 100091, China

## Abstract

Aging is a main risk factor for a number of debilitating diseases and contributes to an increase in mortality. Previous studies have shown that *Ginkgo biloba* extract (EGb) can prevent and treat aging-related diseases, but its pharmacological effects need to be further clarified. This study aimed to propose a network pharmacology-based method to identify the therapeutic pathways of EGb for aging. The active components of EGb and targets of sample chemicals were obtained from the Traditional Chinese Medicine Systems Pharmacology Database and Analysis Platform (TCMSP) database. Information on aging-related genes was obtained from the Human Ageing Genomic Resources database and JenAge Ageing Factor Database. Subsequently, a network containing the interactions between the putative targets of EGb and known therapeutic targets of aging was established, which was used to investigate the pharmacological mechanisms of EGb for aging. A total of 24 active components, 154 targets of active components of EGb, and 308 targets of aging were obtained. Network construction and pathway enrichment were conducted after data integration. The study found that flavonoids (quercetin, luteolin, and kaempferol) and beta-sitosterol may be the main active components of EGb. The top eight candidate targets, namely, PTGS2, PPARG, DPP4, GSK3B, CCNA2, AR, MAPK14, and ESR1, were selected as the main therapeutic targets of EGb. Pathway enrichment results in various pathways were associated with inhibition of oxidative stress, inhibition of inflammation, amelioration of insulin resistance, and regulation of cellular biological processes. Molecular docking results showed that PPARG had better binding capacity with beta-sitosterol, and PTGS2 had better binding capacity with kaempferol and quercetin. The main components of EGb may act on multiple targets, such as PTGS2, PPARG, DPP4, and GSK3B, to regulate multiple pathways, and play an antiaging role by inhibiting oxidative stress, inhibiting inflammation, and ameliorating insulin resistance.

## 1. Introduction

Aging is inevitable, but pathological aging poses a serious threat to human health, reduces the quality of life of elderly individuals, and promotes the development of related diseases [1, 2]. Aging is an extremely important factor that induces many types of cardiovascular and cerebrovascular diseases, such as atherosclerosis, myocardial infarction, stroke, and heart failure [3, 4]. Among them, vascular aging plays a central role in morbidity and mortality [5]. After years of research, researchers have put forward many hypotheses regarding aging mechanisms, such as oxidative stress, genetic determination theory, free radical theory, neuroendocrine theory, and mitochondrial damage theory [4, 6, 7]. Most importantly, increasing oxidative stress, a major characteristic of aging, has been implicated in various age-related diseases [8]. The biological mechanisms of aging are still being explored. As fertility declines and life expectancy increases, the proportion of the aging global population is increasing, and the presence of age-related diseases increases the socioeconomic burden. Therefore, it has become one of the most important issues in studying the biological mechanisms of aging and exploring effective drugs that can delay or reduce the development of age-related diseases and clarify the mechanism. It is believed that many medicinal herbs have antiaging properties, but the mechanisms remain unclear.

The extract of *Ginkgo biloba* (EGb), a well-known medicinal plant, is used to treat many aging-related diseases, such as cardiovascular and neurodegenerative aging-related diseases [9]. Due to its medicinal value, increasing attention has been paid to the basic research and clinical application of EGb. A study found that the chemical composition of EGb is complex, including flavonoids, lactones, polyphenols, alkyl phenolic acids, organic acids, steroids, and trace elements [10]. EGb preparation is a relatively successful case of plant medicine developed by modern science and technology and occupies an important position in the field of antiaging and clinical medication for cardiovascular and cerebrovascular diseases [9, 11]. Previous studies have shown that EGb has certain efficacy in the prevention and treatment of age-related diseases [9, 12–14]. However, the pharmacologic profiles of EGB for aging remain unclear. Therefore, in this study, a network pharmacology method was used to preliminarily explore the molecular mechanism of EGb in aging.

## 2. Materials and Methods

### 2.1. Identification and Screening of Candidate Compounds

The active constituents of EGb were obtained from the Traditional Chinese Medicine Systems Pharmacology Database and Analysis Platform (TCMSP) database (http://lsp.nwsuaf.edu.cn/tcmsp.php). TCMSP is an integrated systems pharmacology platform of Chinese herbal medicines that covers chemicals, targets, and drug targets and pharmacokinetic properties for compounds involving oral bioavailability (OB), drug likeness (DL), blood-brain barrier, intestinal epithelial permeability, and aqueous solubility [15]. In this study, OB and DL were utilized to screen the chemical constituents with OB ≥ 30% and DL ≥ 0.18 simultaneously [16]. In addition, active ingredients with low OB and remarkable efficacy reported in the literature were included.

### 2.2. Potential Targets of Active Components

The validated target proteins of the active components were obtained from the TCMSP database. The target protein name of the active ingredient was converted to the standard target gene name through the UniProt Knowledge Base (UniProtKB, http://www.uniprot.org/). The UniProtKB is the central hub for the collection of functional information on proteins, with accurate, consistent, and rich annotation. The target protein names were inputted into UniProtKB, with the organism restricted to “Homo sapiens,” eventually gaining the official symbol.

### 2.3. Search of Aging-Related Targets and Genes

Aging-related genes were obtained by retrieving data from the Human Ageing Genomic Resources (HAGR) database (http://genomics.senescence.info/) and JenAge Ageing Factor Database (AgeFactDB, http://agefactdb.jenage.de). HAGR is a collection of databases and tools for studying human aging through modern approaches, such as functional genomics, network analysis, systems biology, and evolutionary analysis [17]. The purpose of the AgeFactDB is to collect and integrate aging phenotype and longevity data, and it also includes genes that are homologous to known aging-related genes [18]. The target matching analysis of aging-related genes and EGb was conducted to select the target of EGb acting on aging.

### 2.4. Construction of Networks and Analyses

The Cytoscape software was adopted to visually display and analyze the network structure. It is an open source software platform for visualizing molecular interaction networks and biological pathways and integrating these networks with annotations, gene expression profiles, and other state data [19]. Two networks were constructed in this study as follows. (1) A component-component target network was established by joining the active components of herbs and their corresponding components. (2) Based on the component-component target network, the core network was screened according to the degree value.

### 2.5. Gene Ontology (GO) and Pathway Enrichment Analysis

The Database for Annotation, Visualization, and Integrated Discovery (DAVID) provided a comprehensive set of functional annotation tools to understand the biological meaning behind a large list of genes. DAVID tools were able to identify enriched biological themes, particularly GO terms that involved biological process (BP), cell component (CC), and molecular functions (MFs) and visualized genes on KEGG pathway maps [20]. The target of EGb acting on aging was inputted into the DAVID database with the organism limited to “Homo sapiens.” The dividing value of recognized GO terms was set to FDR < 0.05, and KEGG pathways were set to *P* < 0.05 [21, 22].

### 2.6. Molecular Docking

In the analysis of the component-target action network, it is generally believed that a node with greater degree is the pivot node in the whole network. Therefore, the components of the core network were molecularly docked with the target. The 3D structure of the core components was obtained using PubChem (https://pubchem.ncbi.nlm.nih.gov/), and target proteins were retrieved from the Protein Data Bank (http://www.rcsb.org/pdb). The main pharmacodynamic components of *Ginkgo biloba* were docked with the core target gene by AutoDock. PyMOL was used to display the interaction diagram between receptor proteins and ligand small molecules.

## 3. Results

### 3.1. Identification of the Active Compounds in EGb

A total of 24 active ingredients of EGb were included according to the OB and DL values in the TCMSP database and reported in the literature. The results are shown in Additional file 1. The OB of ginkgolide A was <30%, but it was a common compound in EGb and was shown to prevent and treat Alzheimer's disease, a disease associated with aging [14, 23]. Hence, ginkgolide A was also considered a candidate compound.

### 3.2. Identification of Targets in EGb

By searching the abovementioned databases, 154 targets of active ingredients of EGb (Additional file 2: targets of active ingredients) and 308 targets of aging (Additional file 3: targets of aging) were obtained. Moreover, 28 targets of active components of EGb for antiaging were obtained by integrating the intersection of targets of active ingredients and aging (Additional file 4: potential targets of EGb for antiaging).

### 3.3. Component-Component Target Network Analysis

**Figure 1:**
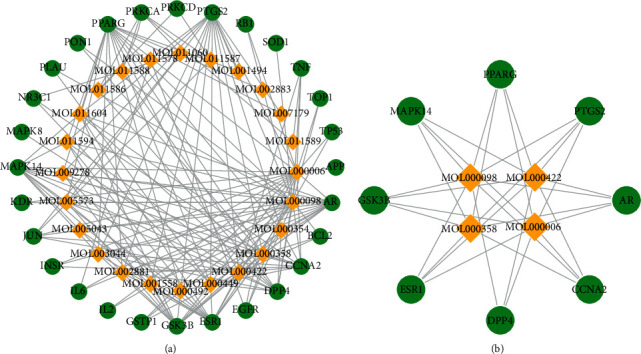
Component-component target network. (a) The network of EGb components and component targets with therapeutic effects against aging. (b) Filter the core network with a degree >10 based on component-component target network. The diamond shape represents the components, and the ellipse represents the target gene.

The 24 active ingredients of EGb and 28 targets of the active components of EGb for antiaging were retrieved. We constructed networks to present the relationship among EGb components and component targets with therapeutic effects against aging. The network consists of 52 nodes and 164 edges (Figure 1(a)). Based on the component-target network, the core network was screened according to the degree. The screening condition was a degree >10. Four compounds, including quercetin (MOL000098), luteolin (MOL000006), kaempferol (MOL000422), and beta-sitosterol (MOL000358), and eight targets, including PTGS2, PPARG, GSK3B, DPP4, CCNA2, AR, MAPK14, and ESR1, had a degree >10 (Figure 1(b)). The results indicated that these four components might play a vital role in the antiaging network of EGb through the abovementioned target genes.

### 3.4. GO and KEGG Pathway Enrichment Analysis

**Figure 2:**
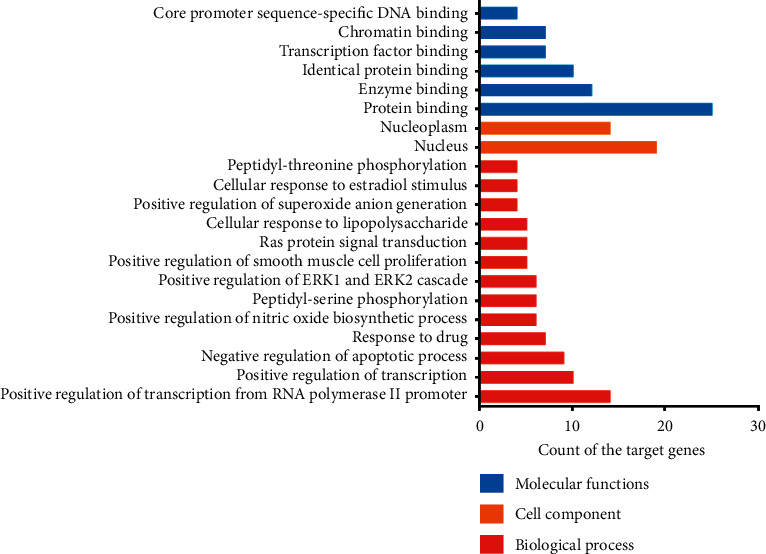
GO enrichment analysis of putative therapeutic targets.

To illustrate the mechanism of EGb in aging, we performed GO and KEGG pathway enrichment analysis for 27 putative therapeutic targets. We identified enrichment results in the related items: 195 BPs, 42 MFs, and 25 CCs. Subsequently, 21 significantly enriched GO terms with FDR <0.05 were determined, including 13 BPs, 2 CCs, and 6 MFs (Figure 2). For BPs, these putative therapeutic targets were mainly enriched in positive regulation of transcription from RNA polymerase II promoter (GO:0045944), positive regulation of nitric oxide biosynthetic process (GO:0045429), positive regulation of transcription, DNA-templated (GO:0045893), positive regulation of superoxide anion generation (GO:0032930), negative regulation of apoptotic process (GO:0043066), peptidyl-serine phosphorylation (GO:0018105), positive regulation of smooth muscle cell proliferation (GO:0048661), Ras protein signal transduction (GO:0007265), response to drug (GO:0042493), positive regulation of extracellular signal-regulated kinase (ERK) 1 and 2 cascade (GO:0070374), cellular response to estradiol stimulus (GO:0071392), cellular response to lipopolysaccharide (GO:0071222), and peptidyl-threonine phosphorylation (GO:0018107). For CCs, the targets were enriched in the nucleoplasm (GO:0005654) and nucleus (GO:0005634). For MFs, the targets were enriched with enzyme binding (GO:0019899), identical protein binding (GO:0042802), transcription factor binding (GO:0008134), protein binding (GO:0005515), chromatin binding (GO:0003682), and core promoter sequence-specific DNA binding (GO:0001046). Therefore, the results indicated that EGb mainly exerted antiaging therapeutic effects by regulating these BPs, MFs, and CCs.

The *X*-axis shows the counts of target genes, and the *Y*-axis shows significantly enriched GO categories of the target genes (FDR < 0.05).

**Figure 3:**
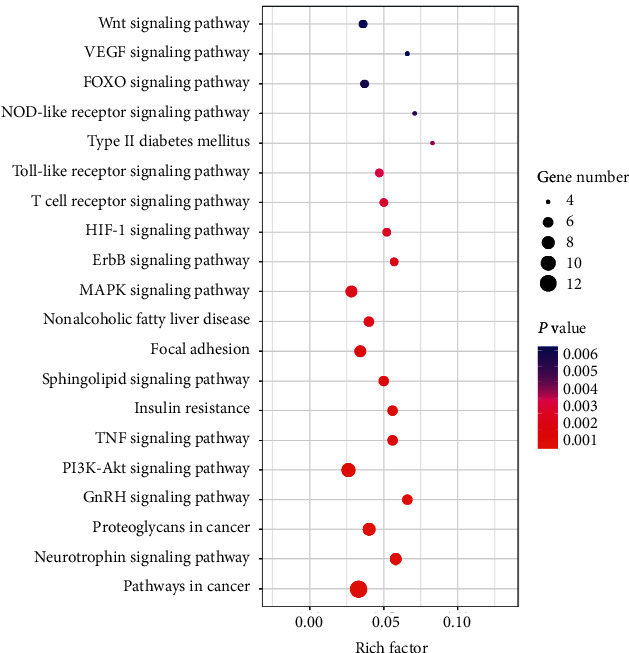
KEGG pathway enrichment analyses of putative therapeutic targets.

We performed KEGG pathway enrichment analysis to examine the pathways for 27 putative therapeutic targets of EGb for antiaging. The top 20 pathways were determined (Figure 3), mainly including pathways in cancer (hsa05200), neurotrophin signaling pathway (hsa04722), proteoglycans in cancer (hsa05205), gonadotropin-releasing hormone (GnRH) signaling pathway (hsa04912), PI3K-Akt signaling pathway (hsa04151), the tumor necrosis factor (TNF) signaling pathway (hsa04668), insulin resistance (hsa04931), sphingolipid signaling pathway (hsa04071), focal adhesion (hsa04510), nonalcoholic fatty liver disease (hsa04932), MAPK signaling pathway (hsa04010), ErbB signaling pathway (hsa04012), HIF-1 signaling pathway (hsa04066), T cell receptor signaling pathway (hsa04660), Toll-like receptor signaling pathway (hsa04620), type II diabetes mellitus (hsa04930), NOD-like receptor signaling pathway (hsa04621), FOXO signaling pathway (hsa04068), VEGF signaling pathway (hsa04370), and Wnt signaling pathway (hsa04310). The results indicate that these pathways might play an important role in the antiaging effect of EGb. It provided research ideas for exploring the targets of EGb for antiaging.

The *Y*-axis represents significant pathways of the target genes, and the *X*-axis shows the rich factor. The rich factor represents the ratio of the number of target genes belonging to a pathway to the number of all annotated genes located in the pathway. A higher rich factor represents a higher enrichment level. The color of the dot corresponds to different *P* values, and the size of the dot reflects the number of target genes expressed in the pathway.

### 3.5. Molecular Docking

Eight proteins were docked with four components using AutoDock, and 20 conformations were obtained. The binding energy scoring and docking parameters are shown in Table 1. A low binding score is prone to interaction. Therefore, the three groups with the lowest scores were selected for composition analysis. The conformations of receptor protein PPARG and beta-sitosterol ligand small molecules, receptor protein PTGS2 and kaempferol ligand small molecules, and receptor protein PTGS2 and quercetin ligand small molecules were constructed.

**Figure 4:**
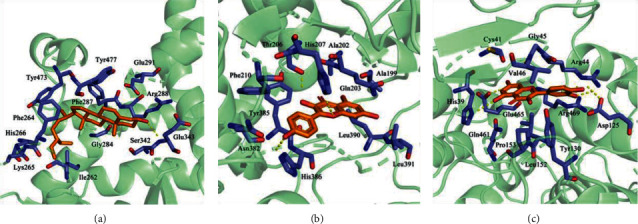
Docking diagram of components and protein molecules. (a) The binding pattern between receptor protein PPARG and beta-sitosterol ligand small molecules. (b) The binding pattern between receptor protein PTGS2 and kaempferol ligand small molecules. (c) The binding pattern between receptor protein PTGS2 and quercetin ligand small molecules.

The binding pattern between receptor protein PPARG and beta-sitosterol ligand small molecules is presented in Figure 4(a). Amino acid residues Glu343 and beta-sitosterol ligand small molecules formed hydrogen bond interactions, and amino acid residues Tyr473, Phe264, His266, Lys265, Ile262, Gly284, Ser342, Arg288, Glu291, Tyr477, and Phe287 and beta-sitosterol ligand small molecules formed hydrophobic interactions.

The binding pattern between the receptor protein PTGS2 and kaempferol ligand small molecules is shown in Figure 4(b). Amino acid residues Thr206, His207, and His386 and kaempferol ligand small molecules formed hydrogen bond interactions, and amino acid residues Ala202, Ala199, Gln203, Leu391, Leu390, Asn382, Tyr385, and Phe210 and kaempferol ligand small molecules formed hydrophobic interactions.

The binding pattern between the receptor protein PTGS2 and quercetin ligand small molecules is shown in Figure 4(c). Amino acid residues Glu465, His39, Arg44, and Asp125 and quercetin ligand small molecules formed hydrogen bond interactions, and amino acid residues Cys41, Gly45, Val46, Arg469, Tyr130, Leu152, Pro153, and Gln461 and quercetin ligand small molecules formed hydrophobic interactions.

## 4. Discussion

**Figure 5:**
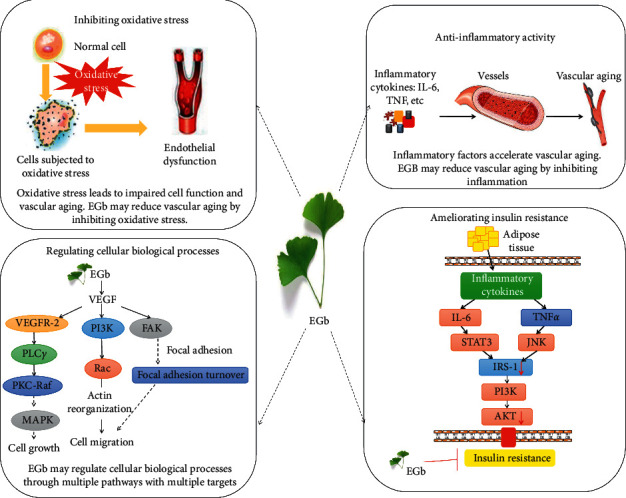
Possible mechanism of EGb for aging.

Previous studies have shown that EGb is effective in the prevention and treatment of aging-related diseases [12–14]. However, the mechanism of its antiaging action needs to be further explored. Therefore, the network pharmacology method was used to predict the possible mechanism and main active components of EGb in delaying aging to provide an innovative idea for further animal experiments. The possible mechanism of EGb for aging is shown in Figure 5. The results of this study also show that various compounds of EGb have antiaging effects. Quercetin, luteolin, kaempferol, and beta-sitosterol may play vital roles in the antiaging network of EGb based on the prediction method of network pharmacology. Quercetin, a polyphenol widely present in nature, has received the most attention in antiaging. Studies have shown that quercetin exerts neuroprotective actions in the aging nervous system by stimulating cellular defenses against oxidative stress, activating sirtuin 1 (SIRT1), and inducing autophagy [24]. Quercetin plays an antiaging role via enhancement of cell proliferation and restoration of heterochromatin architecture [25]. In addition, several evidence-based studies suggest that quercetin is a significant research prospect in the prevention and treatment of vascular aging-related diseases [26, 27]. Luteolin has the potential for antioxidative activity. Luteolin, as an anti-inflammatory and neuroprotective agent, can inhibit the generation of reactive oxygen species via modulation of the AMPK-SIRT1 pathway to prevent the development of age-related disorders [28]. The study also found that luteolin may improve cognitive performance in older mice by inhibiting microglial activation and neuroinflammation [29]. Luteolin may be potentially useful in the prevention of skin aging by inhibiting the UVA-induced production of collagen MMP-1 [30]. A prospective study showed that vegetables and foods rich in kaempferol, lutein, and folate may slow cognitive decline with aging [31]. Kaempferol is an antioxidant and anti-inflammatory agent that regulates the NF-kappaB signaling cascade and inhibits NADPH oxidase activation; hence, it is considered as a potential antiaging agent [32]. Beta-sitosterol may increase the proliferation and stimulate the differentiation of embryonic neural stem cells to prevent neurodevelopmental syndromes and cognitive decline during aging [33]. Beta-sitosterol can extend the lifespan of adult flies, possibly by activating AMP-activated protein kinase (AMPK) [34].

These eight targets, namely, DPP4, GSK3B, CCNA2, AR, ESR1, PTGS2, PPARG, and MAPK14, may play a crucial role in the antiaging network of EGb. DPP4 and GLP-1 had been found to play important roles in oxidative stress, lipid metabolism, insulin resistance, and inflammation. Recently, a study found that the balance between DPP4 and GLP-1 might be a therapeutic target for the management of vascular aging and atherosclerosis in animals [35]. Studies have shown that GSK3B, which acts as a negative regulator in the hormonal control of glucose homeostasis, Wnt signaling, and regulation of transcription factors and microtubules, is closely related to the Healthy Aging Index [36]. Silencing of CCNA2, which controls both the G1/S and G2/M transition phases of the cell cycle, remarkably triggered cellular aging, while CCNA2 overexpression delayed cellular aging [37]. The expression of PTGS, with a particular role in the inflammatory response, was upregulated in diseases related to brain aging [38]. PPARG, a key regulator of adipocyte differentiation and glucose homeostasis, is associated with inflammation [39]. MAPK14 plays important roles in cell proliferation, differentiation, migration, transformation, and programmed cell death [40].

In the GO enrichment analysis, the targets were closely related to positive regulation of transcription from RNA polymerase II promoter, nitric oxide biosynthetic process, smooth muscle cell proliferation, and negative regulation of the apoptotic process. CCs involve the nucleoplasm nucleus. MFs involve the binding of enzymes, proteins, and transcription factors. According to a study by Nakazawa et al., RNA polymerase II ubiquitination provides a two-tier protection mechanism by activating transcription-coupled nucleotide excision repair and, in parallel, processing of DNA damage-stalled RNA polymerase II, which together prevent prolonged transcription arrest and protect against neurodegeneration [41]. Nitric oxide generated through endothelial nitric oxide synthase acts as a gas signaling mediator to promote mitochondrial biogenesis and bioenergetics, with a favorable impact on chronic age-related diseases [42]. There is evidence suggesting that vascular smooth muscle cells accelerate aging and increase the incidence of age-related diseases, such as obesity, diabetes, and atherosclerosis [43, 44]. According to a study by Zhan et al. [45], vascular smooth muscle cells pretreated with the AMPK activator and mammalian target of rapamycin inhibitor could reduce the replication aging of cells. The apoptotic process plays an important role in the aging process [46]. Moorefield et al. [47] used histological methods to detect the morphology, cell composition, and apoptosis of cryptovilli in old (18–22 months) and young (2–4 months) Sox9-EGFP IESC reporter mice, and cleaved caspase-3 staining showed increased apoptotic cells in the crypt and villi of older mice. The study found that some conserved transcription factors, such as the helix-loop-helix transcription factor and forkhead transcription factor (FOXO), control the expression of many autophagy-related genes and have important implications for longevity [48]. A study by Alvarez-Garcia et al. [49] found that the Col2a1Cre and AcanCreER mouse lacking all FOXO isoforms (FOXO 1, 3, and 4) led to severe cell loss in the nucleus pulposus and cartilage endplates, leading to spontaneous degeneration of the intervertebral disk (IVD), which is a prevalent age-associated musculoskeletal disorder, and the findings demonstrate that FOXOs are critical regulators of IVD homeostasis during aging and suggest that maintaining or restoring FOXO expression can delay the onset of IVD degeneration.

KEGG pathway enrichment analyses demonstrated that EGb is involved in the regulation of multiple pathways. Increased oxidative stress and inflammation, including the HIF-1 and TNF signaling pathways, play significant roles in aging, especially vascular aging [50]. The neurotrophin signaling pathway plays an important role in higher-order activities, such as neural development, learning, and memory, and several genes in the pathway are closely related to brain aging [51]. Insulin resistance is strongly associated with type II diabetes and nonalcoholic fatty liver disease, and the elderly population often develops insulin resistance [52]. Oxidative stress, mitochondrial dysfunction, accumulation of intracellular lipid derivatives, and inflammation (via IL-6 and TNF*α*) contribute to decreased activation of signaling molecules, including PI3K and AKT, leading to insulin resistance [53–55]. The absence of sphingomyelin, a second messenger that functions in a variety of cellular signaling pathways, leads to a shortened lifespan in animals, suggesting that sphingolipid signaling may play a role in neuronal function and animal stress response during aging [56]. Focal adhesion, mitogen-activated protein kinase (MAPK) signaling pathway, and ErbB signaling pathway are modules that are involved in cell proliferation, differentiation, and migration [57–59]. Focal adhesion, a signaling molecule associated with cell survival, relies on the interaction between integrins and actin to connect cells to the extracellular matrix [58]. Studies have shown that extracellular matrix protein-cell interactions give rise to target organ damage and inflammatory pathways, leading to calcification or atherosclerosis [1, 60]. The MAPK signaling pathway, including ERK1/2, c-Jun N-terminal kinase, and p38, is closely related to the BP of aging, and the progression of the inflammatory process leads to dysregulation of MAPK, which accelerates cell aging [61]. Inactivation of the ErbB signaling pathway leads to a loss of myocardial protective function during cardiac hypertrophy and onset of early failure [59]. The *T* cell receptor signaling pathway, Toll-like receptor (TLR) signaling pathway, and NOD-like receptor signaling pathway are responsible for generating innate immune responses and play a critical role in inflammation. Toll-like receptors play a significant role in promoting aging adipose tissue inflammation, and the study showed that old TLR4-deficient mice have improved glucose tolerance compared to age-matched wild-type mice [62]. A different set of NOD-like receptors induces caspase-1 activation, and the activated caspase-1 regulates the maturation of the proinflammatory cytokines IL-1B and IL-18 and drives pyroptosis [63]. The FOXO family regulates the expression of genes involved in apoptosis, cell-cycle control, glucose metabolism, oxidative stress resistance, and longevity. Recent evidence indicates that the FOXO family plays a key role in the self-renewal of adult and embryonic stem cells, which could contribute to tissue regeneration [64]. Vascular endothelial growth factor (VEGF) can stimulate endothelial cell growth, promote angiogenesis, and increase vascular permeability. The decreased angiogenesis associated with the aging of the body is related to the decreased expression of VEGF, and the proliferation ability of vascular endothelial cells in elderly animals is weakened. The addition of VEGF can help restore the proliferation ability of vascular endothelial cells, leading to delayed vascular aging [65]. Wnt proteins are secreted morphogens that are required for basic developmental processes. Overactivation of the Wnt/*β*-catenin signaling pathway is closely related to stem cell aging [66].

## 5. Conclusions

In this study, the active components and mechanism of EGb for aging were analyzed based on network pharmacology. Four compounds, eight targets, and 20 significant pathways in EGb were identified by network analysis, which explained the mechanism of EGb for aging by multiple components, targets, and pathways. Our study found that flavonoids (quercetin, luteolin, and kaempferol) and beta-sitosterol, the main active components of EGb, might slow aging by inhibiting oxidative stress, inhibiting inflammation, ameliorating insulin resistance, and regulating BPs (cell proliferation, differentiation, and migration). Molecular docking results showed that PPARG had better binding capacity with beta-sitosterol, and PTGS2 had better binding capacity with kaempferol and quercetin. However, additional experiments must be conducted to verify these results for more evidence in the future.

## Figures and Tables

**Table 1:** Molecular docking of eight proteins and four components.

Protein	Grid size	Docking score (kcal/mol)			
		Quercetin	Luteolin	Kaempferol	Beta-sitosterol
GSK3B	40 × 40 × 40	−8.1	−8.5	−8.5	−9.0
DPP4	60 × 58 × 48	−8.6	−8.2	−8.2	−8.4
PPARG	40 × 40 × 40	−8.3	−8.3	−8.3	−9.7
CCNA2	40 × 40 × 40	−7.6	−7.7	−7.7	−7.4
AR	40 × 40 × 40	−8.8	−8.7	−8.7	−8.9
PTGS2	40 × 40 × 40	−9.6	−9.4	−9.5	−8.5
ESR1	42 × 44 × 44	−9.2	−9.0	−9.0	−7.9
MAPK14	40 × 40 × 40	−8.4	−8.6	−8.5	−8.3

## Data Availability

The data used to support the study are available from the corresponding author upon request.

## Supplementary Materials

Supplementary Materials Additional file 1: chemical information of main compounds in EGb. Additional file 2: targets of active ingredients. Additional file 3: target of aging. Additional file 4: potential targets of EGb for antiaging.

## References

[1] Lacolley P., Regnault V., Segers P., Laurent S. (2017). Vascular smooth muscle cells and arterial stiffening: relevance in development, aging, and disease. *Physiological Reviews*.

[2] Cleeland C., Pipingas A., Scholey A., White D. (2019). Neurochemical changes in the aging brain: a systematic review. *Neuroscience & Biobehavioral Reviews*.

[3] De Almeida A., Ribeiro T. P., De Medeiros I. A. (2017). Aging: molecular pathways and implications on the cardiovascular system. *Oxidative Medicine and Cellular Longevity*.

[4] Ding A.-J., Zheng S.-Q., Huang X.-B. (2017). Current perspective in the discovery of anti-aging agents from natural products. *Natural Products and Bioprospecting*.

[5] Ungvari Z., Tarantini S., Donato A. J. (2012). Mechanisms of vascular aging. *Circulation Research*.

[6] Hamon M. P., Ahmed E. K., Baraibar M. A. (2019). Proteome oxidative modifications and impairment of specific metabolic pathways during cellular senescence and aging. *Proteomics*.

[7] Moltedo O., Remondelli P., Amodio G. (2019). The mitochondria-endoplasmic reticulum contacts and their critical role in aging and age-associated diseases. *Frontiers in Cell and Developmental Biology*.

[8] Zhang H., Davies K. J. A., Forman H. J. (2015). Oxidative stress response and Nrf_2_ signaling in aging. *Free Radical Biology and Medicine*.

[9] Zuo W., Yan F., Zhang B., Li J., Mei D. (2017). Advances in the studies of Ginkgo biloba leaves extract on aging-related diseases. *Aging and Disease*.

[10] Ude C., Schubert-Zsilavecz M., Wurglics M. (2013). Ginkgo biloba extracts: a review of the pharmacokinetics of the active ingredients. *Clinical Pharmacokinetics*.

[11] Liu Y., Weng W., Gao R. (2019). New insights for cellular and molecular mechanisms of aging and aging-related diseases: herbal medicine as potential therapeutic approach. *Oxidative Medicine and Cellular Longevity*.

[12] Phu H. T., Thuan D. T. B., Nguyen T. H. D., Posadino A. M., Eid A. H., Pintus G. (2020). Herbal medicine for slowing aging and aging-associated conditions: efficacy, mechanisms and safety. *Current Vascular Pharmacology*.

[13] Thancharoen O., Limwattananon C., Waleekhachonloet O., Rattanachotphanit T., Limwattananon P., Limpawattana P. (2019). Ginkgo biloba extract (EGb761), cholinesterase inhibitors, and memantine for the treatment of mild-to-moderate Alzheimer’s disease: a network meta-analysis. *Drugs & Aging*.

[14] Kuo L.-C., Song Y.-Q., Yao C.-A. (2019). Ginkgolide A prevents the amyloid-*β*-induced depolarization of cortical neurons. *Journal of Agricultural and Food Chemistry*.

[15] Ru J., Li P., Wang J. (2014). TCMSP: a database of systems pharmacology for drug discovery from herbal medicines. *Journal of Cheminformatics*.

[16] Huang J., Li L., Cheung F. (2017). Network pharmacology-based approach to investigate the analgesic efficacy and molecular targets of xuangui dropping pill for treating primary dysmenorrhea. *Evidence-Based Complementary and Alternative Medicine*.

[17] Tacutu R., Craig T., Budovsky A. (2013). Human ageing genomic resources: integrated databases and tools for the biology and genetics of ageing. *Nucleic Acids Research*.

[18] Hühne R., Thalheim T., Sühnel J. (2014). AgeFactDB-the JenAge ageing factor database-towards data integration in ageing research. *Nucleic Acids Research*.

[19] Doncheva N. T., Morris J. H., Gorodkin J., Jensen L. J. (2019). Cytoscape StringApp: network analysis and visualization of proteomics data. *Journal of Proteome Research*.

[20] Huang D. W., Sherman B. T., Lempicki R. A. (2009). Systematic and integrative analysis of large gene lists using DAVID bioinformatics resources. *Nature Protocols*.

[21] Gorski M., Van Der Most P. J., Teumer A. (2017). 1000 Genomes-based meta-analysis identifies 10 novel loci for kidney function. *Scientific Reports*.

[22] li Y., Li Y., Lu W. (2018). Integrated network pharmacology and metabolomics analysis of the therapeutic effects of Zi Dian Fang on immune thrombocytopenic purpura. *Frontiers in Pharmacology*.

[23] Chen Y., Wang C., Hu M. (2012). Effects of ginkgolide A on okadaic acid-induced tau hyperphosphorylation and the PI3K-Akt signaling pathway in N2a cells. *Planta Medica*.

[24] Costa L. G., Garrick J. M., Roquè P. J. (2016). Mechanisms of neuroprotection by quercetin: counteracting oxidative stress and more. *Oxidative Medicine and Cellular Longevity*.

[25] Geng L., Liu Z., Zhang W. (2019). Chemical screen identifies a geroprotective role of quercetin in premature aging. *Protein & Cell*.

[26] Kim S. G., Sung J. Y., Kim J.-R., Choi H. C. (2020). Quercetin-induced apoptosis ameliorates vascular smooth muscle cell senescence through AMP-activated protein kinase signaling pathway. *The Korean Journal of Physiology & Pharmacology*.

[27] Patel R. V., Mistry B. M., Shinde S. K., Syed R., Singh V., Shin H.-S. (2018). Therapeutic potential of quercetin as a cardiovascular agent. *European Journal of Medicinal Chemistry*.

[28] Sung B., Chung J. W., Bae H. R., Choi J. S., Kim C. M., Kim N. D. (2015). Humulus japonicus extract exhibits antioxidative and anti-aging effects via modulation of the AMPK-SIRT1 pathway. *Experimental and Therapeutic Medicine*.

[29] Burton M. D., Rytych J. L., Amin R., Johnson R. W. (2016). Dietary luteolin reduces proinflammatory microglia in the brain of senescent mice. *Rejuvenation Research*.

[30] Hwang Y. P., Oh K. N., Yun H. J., Jeong H. G. (2011). The flavonoids apigenin and luteolin suppress ultraviolet A-induced matrix metalloproteinase-1 expression via MAPKs and AP-1-dependent signaling in HaCaT cells. *Journal of Dermatological Science*.

[31] Morris M. C., Wang Y., Barnes L. L., Bennett D. A., Dawson-Hughes B., Booth S. L. (2018). Nutrients and bioactives in green leafy vegetables and cognitive decline. *Neurology*.

[32] Kim J. M., Lee E. K., Kim D. H., Yu B. P., Chung H. Y. (2010). Kaempferol modulates pro-inflammatory NF-*κ*B activation by suppressing advanced glycation endproducts-induced NADPH oxidase. *Age*.

[33] Mahmoudi R., Ghareghani M., Zibara K. (2019). Alyssum homolocarpum seed oil (AHSO), containing natural alpha linolenic acid, stearic acid, myristic acid and *β*-sitosterol, increases proliferation and differentiation of neural stem cells in vitro. *BMC Complementary and Alternative Medicine*.

[34] Lin W.-S., Chen J.-Y., Wang J.-C. (2014). The anti-aging effects of Ludwigia octovalvis on Drosophila melanogaster and SAMP8 mice. *Age*.

[35] Cheng X. W., Narisawa M., Jin E., Yu C., Xu W., Piao L. (2018). Dose rectification of an imbalance between DPP4 and GLP-1 ameliorates chronic stress-related vascular aging and atherosclerosis?. *Clinical and Experimental Pharmacology and Physiology*.

[36] Druley T. E., Wang L., Lin S. J. (2016). Candidate gene resequencing to identify rare, pedigree-specific variants influencing healthy aging phenotypes in the long life family study. *BMC Geriatr*.

[37] Xu S., Wu W., Huang H. (2019). The p53/miRNAs/Ccna2 pathway serves as a novel regulator of cellular senescence: complement of the canonical p53/p21 pathway. *Aging Cell*.

[38] Ryan V. H., Primiani C. T., Rao J. S. (2014). Coordination of gene expression of arachidonic and docosahexaenoic acid cascade enzymes during human brain development and aging. *PLoS One*.

[39] Castellano-Castillo D., Denechaud P. D., Fajas L. (2019). Human adipose tissue H3K4me3 histone mark in adipogenic, lipid metabolism and inflammatory genes is positively associated with BMI and HOMA-IR. *PLoS One*.

[40] Wu W., Zhang W., Choi M. (2019). Vascular smooth muscle-MAPK14 is required for neointimal hyperplasia by suppressing VSMC differentiation and inducing proliferation and inflammation. *Redox Biology*.

[41] Nakazawa Y., Hara Y., Oka Y. (2020). Ubiquitination of DNA damage-stalled RNAPII promotes transcription-coupled repair. *Cell*.

[42] Valerio A., Nisoli E. (2015). Nitric oxide, interorganelle communication, and energy flow: a novel route to slow aging. *Frontiers in Cell and Developmental Biology*.

[43] Monk B. A., George S. J. (2015). The effect of ageing on vascular smooth muscle cell behaviour-a mini-review. *Gerontology*.

[44] Yin Y., Zhou Z., Liu W., Chang Q., Sun G., Dai Y. (2017). Vascular endothelial cells senescence is associated with NOD-like receptor family pyrin domain-containing 3 (NLRP3) inflammasome activation via reactive oxygen species (ROS)/thioredoxin-interacting protein (TXNIP) pathway. *The International Journal of Biochemistry & Cell Biology*.

[45] Zhan J. K., Wang Y. J., Li S. (2018). AMPK/TSC2/mTOR pathway regulates replicative senescence of human vascular smooth muscle cells. *Experimental and Therapeutic Medicine*.

[46] Wu Y., Chen M., Jiang J. (2019). Mitochondrial dysfunction in neurodegenerative diseases and drug targets via apoptotic signaling. *Mitochondrion*.

[47] Moorefield E. C., Andres S. F., Blue R. E. (2017). Aging effects on intestinal homeostasis associated with expansion and dysfunction of intestinal epithelial stem cells. *Aging*.

[48] Lapierre L. R., Kumsta C., Sandri M., Ballabio A., Hansen M. (2015). Transcriptional and epigenetic regulation of autophagy in aging. *Autophagy*.

[49] Alvarez-Garcia O., Matsuzaki T., Olmer M. (2018). FOXO are required for intervertebral disk homeostasis during aging and their deficiency promotes disk degeneration. *Aging Cell*.

[50] Lisanti M. P., Martinez-Outschoorn U. E., Pavlides S. (2011). Accelerated aging in the tumor microenvironment. *Cell Cycle*.

[51] Li J., Zhou Y., Du G., Qin X., Gao L. (2018). Bioinformatic analysis reveals key genes and pathways in aging brain of senescence-accelerated mouse P8 (SAMP8). *CNS & Neurological Disorders-Drug Targets*.

[52] Su T., Xiao Y., Xiao Y. (2019). Bone marrow mesenchymal stem cells-derived exosomal MiR-29b-3p regulates aging-associated insulin resistance. *ACS Nano*.

[53] Martins A. R., Nachbar R. T., Gorjao R. (2012). Mechanisms underlying skeletal muscle insulin resistance induced by fatty acids: importance of the mitochondrial function. *Lipids in Health and Disease*.

[54] Bouzakri K., Roques M., Gual P. (2003). Reduced activation of phosphatidylinositol-3 kinase and increased serine 636 phosphorylation of insulin receptor substrate-1 in primary culture of skeletal muscle cells from patients with type 2 diabetes. *Diabetes*.

[55] Morino K., Petersen K. F., Shulman G. I. (2006). Molecular mechanisms of insulin resistance in humans and their potential links with mitochondrial dysfunction. *Diabetes*.

[56] Chan J. P., Brown J., Hark B. (2017). Loss of sphingosine kinase alters life history traits and locomotor function in Caenorhabditis elegans. *Frontiers in Genetics*.

[57] Peng Q., Deng Z., Pan H., Gu L, Liu O, Tang Z (2018). Mitogen-activated protein kinase signaling pathway in oral cancer. *Oncology Letters*.

[58] Mian M. F., Kang C., Lee S. (2008). Cleavage of focal adhesion kinase is an early marker and modulator of oxidative stress-induced apoptosis. *Chemico-Biological Interactions*.

[59] Wang J., Zhang J., Ding X. (2018). Differential microRNA expression profiles and bioinformatics analysis between young and aging spontaneously hypertensive rats. *International Journal of Molecular Medicine*.

[60] Ben Mahdi M. H., Andrieu V., Pasquier C. (2000). Focal adhesion kinase regulation by oxidative stress in different cell types. *IUBMB Life*.

[61] Cano M., Guerrero-Castilla A., Nabavi S. M., Ayala A., Argüelles S. (2019). Targeting pro-senescence mitogen activated protein kinase (Mapk) enzymes with bioactive natural compounds. *Food and Chemical Toxicology*.

[62] Ghosh A. K., O’Brien M., Mau T., Yung R. (2017). Toll-like receptor 4 (TLR4) deficient mice are protected from adipose tissue inflammation in aging. *Aging*.

[63] Song F., Ma Y., Bai X.-Y., Chen X. (2016). The expression changes of inflammasomes in the aging rat kidneys. *The Journals of Gerontology Series A: Biological Sciences and Medical Sciences*.

[64] Lehtinen M. K., Yuan Z., Boag P. R. (2006). A conserved MST-FOXO signaling pathway mediates oxidative-stress responses and extends life span. *Cell*.

[65] Camici G. G., Savarese G., Akhmedov A., Lüscher T. F. (2015). Molecular mechanism of endothelial and vascular aging: implications for cardiovascular disease. *European Heart Journal*.

[66] Brack A. S., Conboy M. J., Roy S. (2007). Increased Wnt signaling during aging alters muscle stem cell fate and increases fibrosis. *Science*.

