# Supplementary material for: Network Pharmacology-Based Strategy to Investigate the Pharmacological Mechanisms of Ginkgo biloba Extract for Aging
Source: Evid Based Complement Alternat Med. 2020 Jul 27;2020:8508491. doi: 10.1155/2020/8508491 (PMC7403930; doi:10.1155/2020/8508491)
Supplement: Supplementary Materials — Additional file 1: chemical information of main compounds in EGb. Additional file 2: targets of active ingredients. Additional file 3: target of aging. Additional file 4: potential targets of EGb for antiaging. [file 8508491.f1.zip › Additional file/Additional file 1.pdf]

Additional file 1 Chemical information of main compounds in EGb

MOL ID	Molecule name	MW	OB (%)	DL
MOL000006	luteolin	286.25	36.16	0.25
MOL000098	quercetin	302.25	46.43	0.28
MOL000354	isorhamnetin	316.28	49.6	0.31
MOL000358	beta-sitosterol	414.79	36.91	0.75
MOL000422	kaempferol	286.25	41.88	0.24
MOL000449	Stigmasterol	412.77	43.83	0.76
MOL000492	catechin	290.29	54.83	0.24
MOL001494	Mandenol	308.56	42	0.19
MOL001558	sesamin	354.38	56.55	0.83
MOL002881	Diosmetin	300.28	31.14	0.27
MOL002883	Ethyl oleate (NF)	310.58	32.4	0.19
MOL003044	Chryseriol	300.28	35.85	0.27
MOL005043	campest-5-en-3beta-ol	400.76	37.58	0.71
MOL005573	Genkwanin	284.28	37.13	0.24
MOL007179	inolenic acid ethyl este	306.54	46.1	0.2
MOL009278	Laricitrin	332.28	35.38	0.34
MOL011060	ginkgolide A	408.44	13.82	0.74
MOL011586	ginkgolide B	424.44	44.38	0.73
MOL011587	ginkgolide C	440.44	48.33	0.73
MOL011588	ginkgolide J	424.44	44.84	0.73
MOL011589	Ginkgolide M	424.44	49.09	0.75
MOL011594	Isogoycyrol	366.39	40.36	0.83
MOL011604	Syringetin	346.31	36.82	0.37
MOL011578	Bilobalide	326.33	84.42	0.36
