# Supplementary material for: Network Pharmacology-Based Strategy to Investigate the Pharmacological Mechanisms of Ginkgo biloba Extract for Aging
Source: Evid Based Complement Alternat Med. 2020 Jul 27;2020:8508491. doi: 10.1155/2020/8508491 (PMC7403930; doi:10.1155/2020/8508491)
Supplement: Supplementary Materials — Additional file 1: chemical information of main compounds in EGb. Additional file 2: targets of active ingredients. Additional file 3: target of aging. Additional file 4: potential targets of EGb for antiaging. [file 8508491.f1.zip › Additional file/Additional file 2.pdf]

Additional file 2 Targets of active ingredients

Mol ID	Molecular name	Target name	Gene name
MOL000006	luteolin	Amyloid beta A4 protein	APP
MOL000006	luteolin	Androgen receptor	AR
MOL000006	luteolin	Carbonic anhydrase II	CA2
MOL000006	luteolin	Caspase-7	CASP7
MOL000006	luteolin	Cyclin-A2	CCNA2
MOL000006	luteolin	Cell division protein kinase 2	CDPK2
MOL000006	luteolin	Cell division protein kinase 4	CDPK4
MOL000006	luteolin	Dipeptidyl peptidase IV	DPP4
MOL000006	luteolin	Epidermal growth factor receptor	EGFR
MOL000006	luteolin	Estrogen receptor	ESR1
MOL000006	luteolin	Estrogen receptor beta	ESR2
MOL000006	luteolin	Glycogen synthase kinase-3 beta	GSK3B
MOL000006	luteolin	Glutathione S-transferase P	GSTP1
MOL000006	luteolin	Heme oxygenase 1	HMOX1
MOL000006	luteolin	Heat shock protein HSP 90	HSP90AB1
MOL000006	luteolin	Interferon gamma	IFNG
MOL000006	luteolin	Interleukin-2	IL2
MOL000006	luteolin	Interleukin-6	IL6
MOL000006	luteolin	Insulin receptor	INSR
MOL000006	luteolin	Transcription factor AP-1	JUN
MOL000006	luteolin	Vascular endothelial growth factor A	KDR
MOL000006	luteolin	Mitogen-activated protein kinase 1	MAPK1
MOL000006	luteolin	Mitogen-activated protein kinase 14	MAPK14
MOL000006	luteolin	Hepatocyte growth factor receptor	MET
MOL000006	luteolin	Interstitial collagenase	MMP1
MOL000006	luteolin	72 kDa type IV collagenase	MMP2
MOL000006	luteolin	Nuclear receptor coactivator 2	NCOA2
MOL000006	luteolin	Nitric oxide synthase, inducible	NOS2
MOL000006	luteolin	Phosphatidylinositol-4,5-bisphosphate 3-kinase catalytic subunit, gamma isoform	PIK3CD
MOL000006	luteolin	Peroxisome proliferator activated receptor gamma	PPARG
MOL000006	luteolin	Trypsin-1	PRSS1
MOL000006	luteolin	Prostaglandin G/H synthase 1	PTGS1
MOL000006	luteolin	Prostaglandin G/H synthase 2	PTGS2
MOL000006	luteolin	Retinoblastoma-associated protein	RB1
MOL000006	luteolin	Tumor necrosis factor	TNF
MOL000006	luteolin	DNA topoisomerase 1	TOP1
MOL000006	luteolin	Cellular tumor antigen p53	TP53
MOL000006	luteolin	Xanthine dehydrogenase/oxidase	XDH
MOL000098	quercetin	Acetyl-CoA carboxylase 1	ACACB
MOL000098	quercetin	Prostatic acid phosphatase	ACPP
MOL000098	quercetin	Beta-2 adrenergic receptor	ADRB2
MOL000098	quercetin	Aryl hydrocarbon receptor	AHR
MOL000098	quercetin	Aldose reductase	AKR1B1
MOL000098	quercetin	Arachidonate 5-lipoxygenase	ALOX5
MOL000098	quercetin	Androgen receptor	AR
MOL000098	quercetin	Apoptosis regulator Bcl-2	BCL2
MOL000098	quercetin	Carbonic anhydrase II	CA2
MOL000098	quercetin	C-C motif chemokine 2	CCL2

MOL000098	quercetin	Cyclin-A2	CCNA2
MOL000098	quercetin	Cell division protein kinase 2	CDPK2
MOL000098	quercetin	Serine/threonine-protein kinase Chk1	CHEK1
MOL000098	quercetin	Collagen alpha-1(I) chain	COL1A1
MOL000098	quercetin	Collagen alpha-1(III) chain	COL3A1
MOL000098	quercetin	Cathepsin D	CTSD
MOL000098	quercetin	Cytochrome P450 1A2	CYP1A2
MOL000098	quercetin	Cytochrome P450 3A4	CYP3A4
MOL000098	quercetin	Dipeptidyl peptidase IV	DPP4
MOL000098	quercetin	Pro-epidermal growth factor	EGF
MOL000098	quercetin	Epidermal growth factor receptor	EGFR
MOL000098	quercetin	Estrogen receptor	ESR1
MOL000098	quercetin	Estrogen receptor beta	ESR2
MOL000098	quercetin	Coagulation factor VII	F12
MOL000098	quercetin	Tissue factor	F3
MOL000098	quercetin	Gamma-aminobutyric acid receptor subunit alpha-1	GABRA1
MOL000098	quercetin	Glycogen synthase kinase-3 beta	GSK3B
MOL000098	quercetin	Glutathione S-transferase Mu 1	GSTM1
MOL000098	quercetin	Glutathione S-transferase Mu 2	GSTM2
MOL000098	quercetin	Glutathione S-transferase P	GSTP1
MOL000098	quercetin	Heme oxygenase 1	HMOX1
MOL000098	quercetin	Heat shock protein HSP 90	HSP90AB1
MOL000098	quercetin	Acetylcholinesterase	HSPA5
MOL000098	quercetin	Interferon gamma	IFNG
MOL000098	quercetin	Interleukin-1 beta	IL1B
MOL000098	quercetin	Interleukin-2	IL2
MOL000098	quercetin	Interleukin-6	IL6
MOL000098	quercetin	Insulin receptor	INSR
MOL000098	quercetin	Transcription factor AP-1	JUN
MOL000098	quercetin	Potassium voltage-gated channel subfamily H member 2	KCNH2
MOL000098	quercetin	Vascular endothelial growth factor A	KDR
MOL000098	quercetin	Amine oxidase [flavin-containing] B	MAOB
MOL000098	quercetin	Mitogen-activated protein kinase 1	MAPK1
MOL000098	quercetin	Mitogen-activated protein kinase 14	MAPK14
MOL000098	quercetin	Maltase-glucoamylase, intestinal	MGAM
MOL000098	quercetin	Interstitial collagenase	MMP1
MOL000098	quercetin	72 kDa type IV collagenase	MMP2
MOL000098	quercetin	78 kDa glucose-regulated protein	MMP2
MOL000098	quercetin	Stromelysin-1	MMP3
MOL000098	quercetin	Myeloperoxidase	MPO
MOL000098	quercetin	Nuclear receptor coactivator 2	NCOA2
MOL000098	quercetin	Nitric oxide synthase, inducible	NOS2
MOL000098	quercetin	Nitric-oxide synthase, endothelial	NOS3
MOL000098	quercetin	NAD(P)H dehydrogenase [quinone] 1	NQO1
MOL000098	quercetin	Ornithine decarboxylase	ODC1
MOL000098	quercetin	Phosphatidylinositol-4,5-bisphosphate 3-kinase catalytic subunit, gamma isoform	PIK3CD
MOL000098	quercetin	Tissue-type plasminogen activator	PLAT
MOL000098	quercetin	Urokinase-type plasminogen activator	PLAU
MOL000098	quercetin	Serum paraoxonase/arylesterase 1	PON1

MOL000098	quercetin	NADPH--cytochrome P450 reductase	POR
MOL000098	quercetin	Peroxisome proliferator activated receptor gamma	PPARG
MOL000098	quercetin	Trypsin-1	PRSS1
MOL000098	quercetin	Prostaglandin E2 receptor EP3 subtype	PTGER3
MOL000098	quercetin	Prostaglandin G/H synthase 1	PTGS1
MOL000098	quercetin	Prostaglandin G/H synthase 2	PTGS2
MOL000098	quercetin	Retinoblastoma-associated protein	RB1
MOL000098	quercetin	Retinoic acid receptor RXR-alpha	RXRB
MOL000098	quercetin	Sodium channel protein type 5 subunit	SCN5A
MOL000098	quercetin	E-selectin	SELE
MOL000098	quercetin	Superoxide dismutase [Cu-Zn]	SOD1
MOL000098	quercetin	Estrogen sulfotransferase	SULT1E1
MOL000098	quercetin	Thrombomodulin	THBD
MOL000098	quercetin	Tumor necrosis factor	TNF
MOL000098	quercetin	DNA topoisomerase 1	TOP1
MOL000098	quercetin	DNA topoisomerase II	TOP2
MOL000098	quercetin	Cellular tumor antigen p53	TP53
MOL000098	quercetin	Vascular cell adhesion protein 1	VCAM1
MOL000098	quercetin	Xanthine dehydrogenase/oxidase	XDH
MOL000354	isorhamnetin	Acetylcholinesterase	ACHE
MOL000354	isorhamnetin	Aldose reductase	AKR1B1
MOL000354	isorhamnetin	Androgen receptor	AR
MOL000354	isorhamnetin	Carbonic anhydrase II	CA2
MOL000354	isorhamnetin	Calmodulin	CALM1
MOL000354	isorhamnetin	Cyclin-A2	CCNA2
MOL000354	isorhamnetin	Cell division protein kinase 2	CDPK2
MOL000354	isorhamnetin	Serine/threonine-protein kinase Chk1	CHEK1
MOL000354	isorhamnetin	Dipeptidyl peptidase IV	DPP4
MOL000354	isorhamnetin	Estrogen receptor	ESR1
MOL000354	isorhamnetin	Estrogen receptor beta	ESR2
MOL000354	isorhamnetin	Coagulation factor VII	F12
MOL000354	isorhamnetin	Gamma-aminobutyric acid receptor subunit alpha-1	GABRA1
MOL000354	isorhamnetin	Glutamate receptor 2	GRIA2
MOL000354	isorhamnetin	Glycogen synthase kinase-3 beta	GSK3B
MOL000354	isorhamnetin	Heat shock protein HSP 90	HSP90AB1
MOL000354	isorhamnetin	Amine oxidase [flavin-containing] B	MAOB
MOL000354	isorhamnetin	Mitogen-activated protein kinase 14	MAPK14
MOL000354	isorhamnetin	Nuclear receptor coactivator 1	NCOA1
MOL000354	isorhamnetin	Nuclear receptor coactivator 2	NCOA2
MOL000354	isorhamnetin	Nitric oxide synthase, inducible	NOS2
MOL000354	isorhamnetin	Nitric-oxide synthase, endothelial	NOS3
MOL000354	isorhamnetin	Phosphatidylinositol-4,5-bisphosphate 3-kinase catalytic subunit, gamma isoform	PIK3CD
MOL000354	isorhamnetin	Peroxisome proliferator activated receptor delta	PPARD
MOL000354	isorhamnetin	Peroxisome proliferator activated receptor gamma	PPARG
MOL000354	isorhamnetin	Trypsin-1	PRSS1
MOL000354	isorhamnetin	Prostaglandin G/H synthase 1	PTGS1
MOL000354	isorhamnetin	Prostaglandin G/H synthase 2	PTGS2

MOL000354	isorhamnetin	Glycogen phosphorylase, muscle form	PYGM
MOL000354	isorhamnetin	Xanthine dehydrogenase/oxidase	XDH
MOL000358	beta-sitosterol	Acetylcholinesterase	ACHE
MOL000358	beta-sitosterol	Alpha-1A adrenergic receptor	ADRA1A
MOL000358	beta-sitosterol	Alpha-1B adrenergic receptor	ADRA1B
MOL000358	beta-sitosterol	Beta-2 adrenergic receptor	ADRB2
MOL000358	beta-sitosterol	Androgen receptor	AR
MOL000358	beta-sitosterol	Apoptosis regulator Bcl-2	BCL2
MOL000358	beta-sitosterol	Carbonic anhydrase II	CA2
MOL000358	beta-sitosterol	Cyclin-A2	CCNA2
MOL000358	beta-sitosterol	Cell division protein kinase 2	CDPK2
MOL000358	beta-sitosterol	Serine/threonine-protein kinase Chk1	CHEK1
MOL000358	beta-sitosterol	Muscarinic acetylcholine receptor M1	CHRM1
MOL000358	beta-sitosterol	Muscarinic acetylcholine receptor M2	CHRM2
MOL000358	beta-sitosterol	Muscarinic acetylcholine receptor M3	CHRM3
MOL000358	beta-sitosterol	Muscarinic acetylcholine receptor M4	CHRM4
MOL000358	beta-sitosterol	Neuronal acetylcholine receptor subunit alpha-2	CHRNA2
MOL000358	beta-sitosterol	Neuronal acetylcholine receptor protein, alpha-7 chain	CHRNA7
MOL000358	beta-sitosterol	Dipeptidyl peptidase IV	DPP4
MOL000358	beta-sitosterol	Estrogen receptor	ESR1
MOL000358	beta-sitosterol	Estrogen receptor beta	ESR2
MOL000358	beta-sitosterol	Gamma-aminobutyric acid receptor subunit alpha-1	GABRA1
MOL000358	beta-sitosterol	Gamma-aminobutyric-acid receptor alpha-2 subunit	GABRA2
MOL000358	beta-sitosterol	Gamma-aminobutyric-acid receptor alpha-3 subunit	GABRA3
MOL000358	beta-sitosterol	Gamma-aminobutyric-acid receptor alpha-5 subunit	GABRA5
MOL000358	beta-sitosterol	Glycogen synthase kinase-3 beta	GSK3B
MOL000358	beta-sitosterol	Heat shock protein HSP 90	HSP90AB1
MOL000358	beta-sitosterol	5-hydroxytryptamine 2A receptor	HTR2A
MOL000358	beta-sitosterol	Transcription factor AP-1	JUN
MOL000358	beta-sitosterol	Potassium voltage-gated channel subfamily H member 2	KCNH2
MOL000358	beta-sitosterol	Microtubule-associated protein 2	MAP2
MOL000358	beta-sitosterol	Mitogen-activated protein kinase 14	MAPK14
MOL000358	beta-sitosterol	Nuclear receptor coactivator 2	NCOA2
MOL000358	beta-sitosterol	Nitric oxide synthase, inducible	NOS2
MOL000358	beta-sitosterol	Glucocorticoid receptor	NR3C1
MOL000358	beta-sitosterol	Mu-type opioid receptor	OPRM1
MOL000358	beta-sitosterol	CGMP-inhibited 3',5'-cyclic phosphodiesterase A	PDE3A
MOL000358	beta-sitosterol	Progesterone receptor	PGR
MOL000358	beta-sitosterol	Phosphatidylinositol-4,5-bisphosphate 3-kinase catalytic subunit, gamma isoform	PIK3CD
MOL000358	beta-sitosterol	Serum paraoxonase/arylesterase 1	PON1
MOL000358	beta-sitosterol	Peroxisome proliferator activated receptor gamma	PPARG
MOL000358	beta-sitosterol	Trypsin-1	PRSS1

MOL000358	beta-sitosterol	Prostaglandin G/H synthase 1	PTGS1
MOL000358	beta-sitosterol	Prostaglandin G/H synthase 2	PTGS2
MOL000358	beta-sitosterol	Sodium channel protein type 5 subunit	SCN5A
MOL000358	beta-sitosterol	Sodium-dependent serotonin transporter	SLC6A4
MOL000422	kaempferol	Acetylcholinesterase	ACHE
MOL000422	kaempferol	Alpha-1B adrenergic receptor	ADRA1B
MOL000422	kaempferol	Aryl hydrocarbon receptor	AHR
MOL000422	kaempferol	Aldo-keto reductase family 1 member	AKR1C3
MOL000422	kaempferol	Arachidonate 5-lipoxygenase	ALOX5
MOL000422	kaempferol	Androgen receptor	AR
MOL000422	kaempferol	Apoptosis regulator Bcl-2	BCL2
MOL000422	kaempferol	Carbonic anhydrase II	CA2
MOL000422	kaempferol	Calmodulin	CALM1
MOL000422	kaempferol	Cyclin-A2	CCNA2
MOL000422	kaempferol	Cell division protein kinase 2	CDPK2
MOL000422	kaempferol	Serine/threonine-protein kinase Chk1	CHEK1
MOL000422	kaempferol	Muscarinic acetylcholine receptor M1	CHRM1
MOL000422	kaempferol	Muscarinic acetylcholine receptor M2	CHRM2
MOL000422	kaempferol	Cytochrome P450 1A2	CYP1A2
MOL000422	kaempferol	Cytochrome P450 3A4	CYP3A4
MOL000422	kaempferol	Dipeptidyl peptidase IV	DPP4
MOL000422	kaempferol	Estrogen receptor	ESR1
MOL000422	kaempferol	Estrogen receptor beta	ESR2
MOL000422	kaempferol	Coagulation factor VII	F12
MOL000422	kaempferol	Gamma-aminobutyric acid receptor subunit alpha-1	GABRA1
MOL000422	kaempferol	Gamma-aminobutyric-acid receptor alpha-2 subunit	GABRA2
MOL000422	kaempferol	Glycogen synthase kinase-3 beta	GSK3B
MOL000422	kaempferol	Glutathione S-transferase Mu 1	GSTM1
MOL000422	kaempferol	Glutathione S-transferase Mu 2	GSTM2
MOL000422	kaempferol	Glutathione S-transferase P	GSTP1
MOL000422	kaempferol	Heme oxygenase 1	HMOX1
MOL000422	kaempferol	Heat shock protein HSP 90	HSP90AB1
MOL000422	kaempferol	Insulin receptor	INSR
MOL000422	kaempferol	Transcription factor AP-1	JUN
MOL000422	kaempferol	Mitogen-activated protein kinase 14	MAPK14
MOL000422	kaempferol	Mitogen-activated protein kinase 8	MAPK8
MOL000422	kaempferol	Interstitial collagenase	MMP1
MOL000422	kaempferol	Nuclear receptor coactivator 2	NCOA2
MOL000422	kaempferol	Nitric oxide synthase, inducible	NOS2
MOL000422	kaempferol	Nitric-oxide synthase, endothelial	NOS3
MOL000422	kaempferol	Progesterone receptor	PGR
MOL000422	kaempferol	Phosphatidylinositol-4,5-bisphosphate 3-kinase catalytic subunit, gamma isoform	PIK3CD
MOL000422	kaempferol	Peroxisome proliferator activated receptor gamma	PPARG
MOL000422	kaempferol	Serine/threonine-protein phosphatase 2B catalytic subunit alpha isoform	PPP3CA
MOL000422	kaempferol	Trypsin-1	PRSS1
MOL000422	kaempferol	Prostaglandin G/H synthase 1	PTGS1
MOL000422	kaempferol	Prostaglandin G/H synthase 2	PTGS2

MOL000422	kaempferol	E-selectin	SELE
MOL000422	kaempferol	Sodium-dependent noradrenaline	SLC6A2
MOL000422	kaempferol	Tumor necrosis factor	TNF
MOL000422	kaempferol	DNA topoisomerase II	TOP2
MOL000422	kaempferol	Vascular cell adhesion protein 1	VCAM1
MOL000422	kaempferol	Xanthine dehydrogenase/oxidase	XDH
MOL000449	Stigmasterol	Acetylcholinesterase	ACHE
MOL000449	Stigmasterol	Alcohol dehydrogenase 1C	ADH1C
MOL000449	Stigmasterol	Alpha-1A adrenergic receptor	ADRA1A
MOL000449	Stigmasterol	Alpha-1B adrenergic receptor	ADRA1B
MOL000449	Stigmasterol	Alpha-2A adrenergic receptor	ADRA2A
MOL000449	Stigmasterol	Beta-1 adrenergic receptor	ADRB1
MOL000449	Stigmasterol	Beta-2 adrenergic receptor	ADRB2
MOL000449	Stigmasterol	Aldose reductase	AKR1B1
MOL000449	Stigmasterol	Androgen receptor	AR
MOL000449	Stigmasterol	Carbonic anhydrase II	CA2
MOL000449	Stigmasterol	Cell division protein kinase 2	CDPK2
MOL000449	Stigmasterol	Muscarinic acetylcholine receptor M1	CHRM1
MOL000449	Stigmasterol	Muscarinic acetylcholine receptor M2	CHRM2
MOL000449	Stigmasterol	Muscarinic acetylcholine receptor M3	CHRM3
MOL000449	Stigmasterol	Neuronal acetylcholine receptor protein, alpha-7 chain	CHRNA7
MOL000449	Stigmasterol	Chymotrypsinogen B	CTRB1
MOL000449	Stigmasterol	Dipeptidyl peptidase IV	DPP4
MOL000449	Stigmasterol	Estrogen receptor	ESR1
MOL000449	Stigmasterol	Gamma-aminobutyric acid receptor subunit alpha-1	GABRA1
MOL000449	Stigmasterol	Gamma-aminobutyric-acid receptor alpha-3 subunit	GABRA3
MOL000449	Stigmasterol	5-hydroxytryptamine 2A receptor	HTR2A
MOL000449	Stigmasterol	Ig gamma-1 chain C region	IGHG1
MOL000449	Stigmasterol	Leukotriene A-4 hydrolase	LTA4H
MOL000449	Stigmasterol	Amine oxidase [flavin-containing] A	MAOA
MOL000449	Stigmasterol	Amine oxidase [flavin-containing] B	MAOB
MOL000449	Stigmasterol	Nuclear receptor coactivator 1	NCOA1
MOL000449	Stigmasterol	Nuclear receptor coactivator 2	NCOA2
MOL000449	Stigmasterol	Nitric oxide synthase, inducible	NOS2
MOL000449	Stigmasterol	Nitric-oxide synthase, endothelial	NOS3
MOL000449	Stigmasterol	Glucocorticoid receptor	NR3C1
MOL000449	Stigmasterol	Mineralocorticoid receptor	NR3C2
MOL000449	Stigmasterol	Progesterone receptor	PGR
MOL000449	Stigmasterol	Urokinase-type plasminogen activator	PLAU
MOL000449	Stigmasterol	Peroxisome proliferator activated receptor gamma	PPARG
MOL000449	Stigmasterol	Trypsin-1	PRSS1
MOL000449	Stigmasterol	Prostaglandin G/H synthase 1	PTGS1
MOL000449	Stigmasterol	Prostaglandin G/H synthase 2	PTGS2
MOL000449	Stigmasterol	Retinoic acid receptor RXR-alpha	RXRB
MOL000449	Stigmasterol	Sodium channel protein type 5 subunit	SCN5A
MOL000449	Stigmasterol	Sodium-dependent noradrenaline	SLC6A2
MOL000449	Stigmasterol	Sodium-dependent dopamine transporter	SLC6A3
MOL000492	catechin	Androgen receptor	AR

MOL000492	catechin	Carbonic anhydrase II	CA2
MOL000492	catechin	Calmodulin	CALM1
MOL000492	catechin	Cyclin-A2	CCNA2
MOL000492	catechin	Cell division protein kinase 2	CDPK2
MOL000492	catechin	Serine/threonine-protein kinase Chk1	CHEK1
MOL000492	catechin	Dipeptidyl peptidase IV	DPP4
MOL000492	catechin	Estrogen receptor	ESR1
MOL000492	catechin	Estrogen receptor beta	ESR2
MOL000492	catechin	Glycogen synthase kinase-3 beta	GSK3B
MOL000492	catechin	Heat shock protein HSP 90	HSP90AB1
MOL000492	catechin	Mitogen-activated protein kinase 14	MAPK14
MOL000492	catechin	Nuclear receptor coactivator 2	NCOA2
MOL000492	catechin	Nitric oxide synthase, inducible	NOS2
MOL000492	catechin	Peroxisome proliferator activated receptor gamma	PPARG
MOL000492	catechin	Prostaglandin G/H synthase 1	PTGS1
MOL000492	catechin	Prostaglandin G/H synthase 2	PTGS2
MOL000492	catechin	Retinoic acid receptor RXR-alpha	RXRB
MOL001494	Mandenol	Acetylcholinesterase	ACHE
MOL001494	Mandenol	Dipeptidyl peptidase IV	DPP4
MOL001494	Mandenol	Nuclear receptor coactivator 2	NCOA2
MOL001494	Mandenol	Nitric-oxide synthase, endothelial	NOS3
MOL001494	Mandenol	Peroxisome proliferator activated receptor gamma	PPARG
MOL001494	Mandenol	Prostaglandin G/H synthase 1	PTGS1
MOL001494	Mandenol	Prostaglandin G/H synthase 2	PTGS2
MOL001558	sesamin	Acetyl-CoA carboxylase 1	ACACB
MOL001558	sesamin	Medium-chain specific acyl-CoA dehydrogenase, mitochondrial	ACADM
MOL001558	sesamin	Acetylcholinesterase	ACHE
MOL001558	sesamin	Peroxisomal acyl-coenzyme A oxidase 1	ACOX1
MOL001558	sesamin	Androgen receptor	AR
MOL001558	sesamin	Cytochrome P450 2B6	CYP2B6
MOL001558	sesamin	2,4-dienoyl-CoA reductase,	DECR1
MOL001558	sesamin	Dipeptidyl peptidase IV	DPP4
MOL001558	sesamin	Endothelin-converting enzyme 1	ECE1
MOL001558	sesamin	Peroxisomal bifunctional enzyme	EHHADH
MOL001558	sesamin	Estrogen receptor	ESR1
MOL001558	sesamin	Estrogen receptor beta	ESR2
MOL001558	sesamin	Fatty acid synthase	FASN
MOL001558	sesamin	Glycogen synthase kinase-3 beta	GSK3B
MOL001558	sesamin	Mitogen-activated protein kinase 14	MAPK14
MOL001558	sesamin	Nitric oxide synthase, inducible	NOS2
MOL001558	sesamin	Trypsin-1	PRSS1
MOL001558	sesamin	Prostaglandin G/H synthase 2	PTGS2
MOL001558	sesamin	Sodium channel protein type 5 subunit	SCN5A
MOL002881	Diosmetin	Androgen receptor	AR
MOL002881	Diosmetin	Carbonic anhydrase II	CA2
MOL002881	Diosmetin	Calmodulin	CALM1
MOL002881	Diosmetin	Cyclin-A2	CCNA2
MOL002881	Diosmetin	Cell division protein kinase 2	CDPK2
MOL002881	Diosmetin	Serine/threonine-protein kinase Chk1	CHEK1

MOL002881	Diosmetin	Dipeptidyl peptidase IV	DPP4
MOL002881	Diosmetin	Dipeptidyl peptidase 4	DPP4
MOL002881	Diosmetin	Estrogen receptor	ESR1
MOL002881	Diosmetin	Estrogen receptor beta	ESR2
MOL002881	Diosmetin	Glycogen synthase kinase-3 beta	GSK3B
MOL002881	Diosmetin	Heat shock protein HSP 90	HSP90AB1
MOL002881	Diosmetin	Mitogen-activated protein kinase 14	MAPK14
MOL002881	Diosmetin	Nuclear receptor coactivator 1	NCOA1
MOL002881	Diosmetin	Nuclear receptor coactivator 2	NCOA2
MOL002881	Diosmetin	Nitric oxide synthase, inducible	NOS2
MOL002881	Diosmetin	Peroxisome proliferator activated receptor gamma	PPARG
MOL002881	Diosmetin	Trypsin-1	PRSS1
MOL002881	Diosmetin	Prostaglandin G/H synthase 1	PTGS1
MOL002881	Diosmetin	Prostaglandin G/H synthase 2	PTGS2
MOL002883	Ethyl oleate (NF)	Acetylcholinesterase	ACHE
MOL002883	Ethyl oleate (NF)	Nuclear receptor coactivator 2	NCOA2
MOL002883	Ethyl oleate (NF)	Nitric-oxide synthase, endothelial	NOS3
MOL002883	Ethyl oleate (NF)	Peroxisome proliferator activated receptor gamma	PPARG
MOL003044	Chryseriol	Androgen receptor	AR
MOL003044	Chryseriol	Carbonic anhydrase II	CA2
MOL003044	Chryseriol	Calmodulin	CALM1
MOL003044	Chryseriol	Cyclin-A2	CCNA2
MOL003044	Chryseriol	Cell division protein kinase 2	CDPK2
MOL003044	Chryseriol	Serine/threonine-protein kinase Chk1	CHEK1
MOL003044	Chryseriol	Dipeptidyl peptidase IV	DPP4
MOL003044	Chryseriol	Dipeptidyl peptidase 4	DPP4
MOL003044	Chryseriol	Estrogen receptor	ESR1
MOL003044	Chryseriol	Estrogen receptor beta	ESR2
MOL003044	Chryseriol	Glycogen synthase kinase-3 beta	GSK3B
MOL003044	Chryseriol	Heat shock protein HSP 90	HSP90AB1
MOL003044	Chryseriol	Mitogen-activated protein kinase 14	MAPK14
MOL003044	Chryseriol	Nuclear receptor coactivator 1	NCOA1
MOL003044	Chryseriol	Nuclear receptor coactivator 2	NCOA2
MOL003044	Chryseriol	Nitric oxide synthase, inducible	NOS2
MOL003044	Chryseriol	Phosphatidylinositol-4,5-bisphosphate 3-kinase catalytic subunit, gamma isoform	PIK3CD
MOL003044	Chryseriol	Peroxisome proliferator activated receptor gamma	PPARG
MOL003044	Chryseriol	Trypsin-1	PRSS1
MOL003044	Chryseriol	Prostaglandin G/H synthase 1	PTGS1
MOL003044	Chryseriol	Prostaglandin G/H synthase 2	PTGS2
MOL005043	ımpest-5-en-3beta-	Androgen receptor	AR
MOL005043	ımpest-5-en-3beta-	Estrogen receptor	ESR1
MOL005043	ımpest-5-en-3beta-	Progesterone receptor	PGR
MOL005573	Genkwanin	Androgen receptor	AR
MOL005573	Genkwanin	Carbonic anhydrase II	CA2
MOL005573	Genkwanin	Calmodulin	CALM1
MOL005573	Genkwanin	Cyclin-A2	CCNA2
MOL005573	Genkwanin	Cell division protein kinase 2	CDPK2
MOL005573	Genkwanin	Serine/threonine-protein kinase Chk1	CHEK1

MOL005573	Genkwanin	Dipeptidyl peptidase IV	DPP4
MOL005573	Genkwanin	Estrogen receptor	ESR1
MOL005573	Genkwanin	Estrogen receptor beta	ESR2
MOL005573	Genkwanin	Glycogen synthase kinase-3 beta	GSK3B
MOL005573	Genkwanin	Heat shock protein HSP 90	HSP90AB1
MOL005573	Genkwanin	Mitogen-activated protein kinase 14	MAPK14
MOL005573	Genkwanin	Nuclear receptor coactivator 1	NCOA1
MOL005573	Genkwanin	Nuclear receptor coactivator 2	NCOA2
MOL005573	Genkwanin	Nitric oxide synthase, inducible	NOS2
MOL005573	Genkwanin	Peroxisome proliferator activated receptor gamma	PPARG
MOL005573	Genkwanin	Trypsin-1	PRSS1
MOL005573	Genkwanin	Prostaglandin G/H synthase 1	PTGS1
MOL005573	Genkwanin	Prostaglandin G/H synthase 2	PTGS2
MOL005573	Genkwanin	Retinoic acid receptor RXR-alpha	RXRB
MOL007179	olenic acid ethyl e	Acetylcholinesterase	ACHE
MOL007179	olenic acid ethyl e	Dipeptidyl peptidase IV	DPP4
MOL007179	olenic acid ethyl e	Nitric-oxide synthase, endothelial	NOS3
MOL007179	olenic acid ethyl e	Peroxisome proliferator activated receptor gamma	PPARG
MOL007179	olenic acid ethyl e	Prostaglandin G/H synthase 1	PTGS1
MOL007179	olenic acid ethyl e	Prostaglandin G/H synthase 2	PTGS2
MOL009278	Laricitrin	Androgen receptor	AR
MOL009278	Laricitrin	Carbonic anhydrase II	CA2
MOL009278	Laricitrin	Calmodulin	CALM1
MOL009278	Laricitrin	Cyclin-A2	CCNA2
MOL009278	Laricitrin	Cell division protein kinase 2	CDPK2
MOL009278	Laricitrin	Dipeptidyl peptidase IV	DPP4
MOL009278	Laricitrin	Estrogen receptor	ESR1
MOL009278	Laricitrin	Estrogen receptor beta	ESR2
MOL009278	Laricitrin	Glycogen synthase kinase-3 beta	GSK3B
MOL009278	Laricitrin	Heat shock protein HSP 90	HSP90AB1
MOL009278	Laricitrin	Mitogen-activated protein kinase 14	MAPK14
MOL009278	Laricitrin	Nuclear receptor coactivator 2	NCOA2
MOL009278	Laricitrin	Nitric oxide synthase, inducible	NOS2
MOL009278	Laricitrin	Phosphatidylinositol-4,5-bisphosphate 3-kinase catalytic subunit, gamma isoform	PIK3CD
MOL009278	Laricitrin	Peroxisome proliferator activated receptor gamma	PPARG
MOL009278	Laricitrin	Trypsin-1	PRSS1
MOL009278	Laricitrin	Prostaglandin G/H synthase 1	PTGS1
MOL009278	Laricitrin	Prostaglandin G/H synthase 2	PTGS2
MOL009278	Laricitrin	DNA topoisomerase II	TOP2
MOL011060	ginkgolide A	Adenosine deaminase	ADA
MOL011060	ginkgolide A	Sodium/potassium-transporting ATPase subunit alpha-4	ATP1A4
MOL011060	ginkgolide A	Bromodomain adjacent to zinc finger domain protein 2B	BAZ2B
MOL011060	ginkgolide A	Glycine receptor subunit alpha-1	GLRA1
MOL011060	ginkgolide A	Glycine receptor subunit alpha-2	GLRA2
MOL011060	ginkgolide A	Glycine receptor subunit alpha-3	GLRA3
MOL011060	ginkgolide A	Glycine receptor subunit beta	GLRB

MOL011060	ginkgolide A	Corticosteroid 11-beta-dehydrogenase isozyme 2	HSD11B2
MOL011060	ginkgolide A	Transcription factor AP-1	JUN
MOL011060	ginkgolide A	Malonyl-CoA decarboxylase,	MLYCD
MOL011060	ginkgolide A	Glucocorticoid receptor	NR3C1
MOL011060	ginkgolide A	Mineralocorticoid receptor	NR3C2
MOL011060	ginkgolide A	Paired box protein Pax-8	PAX8
MOL011060	ginkgolide A	Pyruvate dehydrogenase (acetyl-transferring)] kinase isozyme 2, mitochondrial	PDK2
MOL011060	ginkgolide A	Pyruvate dehydrogenase (acetyl-transferring)] kinase isozyme 3, mitochondrial	PDK3
MOL011060	ginkgolide A	Pyruvate dehydrogenase (acetyl-transferring)] kinase isozyme 4, mitochondrial	PDK4
MOL011060	ginkgolide A	Protein kinase C alpha type	PRKCA
MOL011060	ginkgolide A	Platelet-activating factor receptor	PTAFR
MOL011578	Bilobalide	Adenosine deaminase	ADA
MOL011578	Bilobalide	ıymotrypsin-like elastase family member [illegible]	CELA2A
MOL011578	Bilobalide	Glycine receptor subunit alpha-2	GLRA2
MOL011578	Bilobalide	Geminin	GMNN
MOL011578	Bilobalide	ɪalonyl-CoA decarboxylase, mitochondri[illegible]	MLYCD
MOL011578	Bilobalide	Paired box protein Pax-8	PAX8
MOL011578	Bilobalide	Sex hormone-binding globulin	SHBG
MOL011578	Bilobalide	Thymidine kinase	TDK
MOL011586	ginkgolide B	Adenosine deaminase	ADA
MOL011586	ginkgolide B	Adenylate cyclase type 1	ADCY1
MOL011586	ginkgolide B	Sodium/potassium-transporting ATPase subunit alpha-4	ATP1A4
MOL011586	ginkgolide B	Chymotrypsin-like elastase family member 2A	CELA2A
MOL011586	ginkgolide B	Dipeptidyl peptidase 4	DPP4
MOL011586	ginkgolide B	Lysosomal alpha-glucosidase	GAA
MOL011586	ginkgolide B	Glycine receptor subunit alpha-1	GLRA1
MOL011586	ginkgolide B	Glycine receptor subunit alpha-2	GLRA2
MOL011586	ginkgolide B	Glycine receptor subunit alpha-3	GLRA3
MOL011586	ginkgolide B	Glycine receptor subunit beta	GLRB
MOL011586	ginkgolide B	Corticosteroid 11-beta-dehydrogenase isozyme 2	HSD11B2
MOL011586	ginkgolide B	Transcription factor AP-1	JUN
MOL011586	ginkgolide B	Nuclear receptor subfamily 1 group I member 2	NR1I2
MOL011586	ginkgolide B	Mineralocorticoid receptor	NR3C2
MOL011586	ginkgolide B	Paired box protein Pax-8	PAX8
MOL011586	ginkgolide B	Phospholipase A2	PLA2G1B
MOL011586	ginkgolide B	Protein FimH	PRKCA
MOL011586	ginkgolide B	Protein kinase C alpha type	PRKCA
MOL011586	ginkgolide B	Protein kinase C beta type	PRKCB
MOL011586	ginkgolide B	Protein kinase C delta type	PRKCD
MOL011586	ginkgolide B	Protein kinase C epsilon type	PRKCE
MOL011586	ginkgolide B	Platelet-activating factor receptor	PTAFR

MOL011586	ginkgolide B	Serine/threonine-protein kinase D3	SIK1
MOL011587	ginkgolide C	Transcription factor AP-1	JUN
MOL011587	ginkgolide C	Protein kinase C alpha type	PRKCA
MOL011587	ginkgolide C	Protein kinase C delta type	PRKCD
MOL011588	ginkgolide J	Dipeptidyl peptidase 4	DPP4
MOL011588	ginkgolide J	Transcription factor AP-1	JUN
MOL011588	ginkgolide J	Protein kinase C alpha type	PRKCA
MOL011589	Ginkgolide M	Adenosine deaminase	ADA
MOL011589	Ginkgolide M	Adenylate cyclase type 1	ADCY1
MOL011589	Ginkgolide M	Sodium/potassium-transporting ATPase subunit alpha-4	ATP1A4
MOL011589	Ginkgolide M	Chymotrypsin-like elastase family member 2A	CELA2A
MOL011589	Ginkgolide M	Lysosomal alpha-glucosidase	GAA
MOL011589	Ginkgolide M	Glycine receptor subunit alpha-1	GLRA1
MOL011589	Ginkgolide M	Glycine receptor subunit alpha-2	GLRA2
MOL011589	Ginkgolide M	Glycine receptor subunit alpha-3	GLRA3
MOL011589	Ginkgolide M	Glycine receptor subunit beta	GLRB
MOL011589	Ginkgolide M	Corticosteroid 11-beta-dehydrogenase isozyme 2	HSD11B2
MOL011589	Ginkgolide M	Indoleamine 2,3-dioxygenase 1	IDO1
MOL011589	Ginkgolide M	Malonyl-CoA decarboxylase,	MLYCD
MOL011589	Ginkgolide M	Matrilysin	MMP7
MOL011589	Ginkgolide M	Nuclear receptor subfamily 1 group I member 2	NR1I2
MOL011589	Ginkgolide M	Mineralocorticoid receptor	NR3C2
MOL011589	Ginkgolide M	Paired box protein Pax-8	PAX8
MOL011589	Ginkgolide M	Phospholipase A2	PLA2G1B
MOL011589	Ginkgolide M	Protein kinase C alpha type	PRKCA
MOL011589	Ginkgolide M	Protein kinase C beta type	PRKCB
MOL011589	Ginkgolide M	Protein kinase C epsilon type	PRKCE
MOL011589	Ginkgolide M	Platelet-activating factor receptor	PTAFR
MOL011589	Ginkgolide M	Tumor necrosis factor	TNF
MOL011594	Isogoycyrol	Androgen receptor	AR
MOL011594	Isogoycyrol	Cell division protein kinase 2	CDPK2
MOL011594	Isogoycyrol	Dipeptidyl peptidase IV	DPP4
MOL011594	Isogoycyrol	Dipeptidyl peptidase 4	DPP4
MOL011594	Isogoycyrol	Estrogen receptor	ESR1
MOL011594	Isogoycyrol	Estrogen receptor beta	ESR2
MOL011594	Isogoycyrol	Glycogen synthase kinase-3 beta	GSK3B
MOL011594	Isogoycyrol	Vascular endothelial growth factor	KDR
MOL011594	Isogoycyrol	Mitogen-activated protein kinase 14	MAPK14
MOL011594	Isogoycyrol	Nitric oxide synthase, inducible	NOS2
MOL011594	Isogoycyrol	Peroxisome proliferator activated receptor gamma	PPARG
MOL011594	Isogoycyrol	Prostaglandin G/H synthase 1	PTGS1
MOL011594	Isogoycyrol	Prostaglandin G/H synthase 2	PTGS2
MOL011604	Syringetin	Androgen receptor	AR
MOL011604	Syringetin	Carbonic anhydrase II	CA2
MOL011604	Syringetin	Calmodulin	CALM1
MOL011604	Syringetin	Cyclin-A2	CCNA2
MOL011604	Syringetin	Cell division protein kinase 2	CDPK2

MOL011604	Syringetin	Dipeptidyl peptidase IV	DPP4
MOL011604	Syringetin	Dipeptidyl peptidase 4	DPP4
MOL011604	Syringetin	Estrogen receptor	ESR1
MOL011604	Syringetin	Estrogen receptor beta	ESR2
MOL011604	Syringetin	Coagulation factor VII	F12
MOL011604	Syringetin	Glycogen synthase kinase-3 beta	GSK3B
MOL011604	Syringetin	Heat shock protein HSP 90	HSP90AB1
MOL011604	Syringetin	Mitogen-activated protein kinase 14	MAPK14
MOL011604	Syringetin	Nuclear receptor coactivator 2	NCOA2
MOL011604	Syringetin	Nitric oxide synthase, inducible	NOS2
MOL011604	Syringetin	Peroxisome proliferator activated receptor gamma	PPARG
MOL011604	Syringetin	Trypsin-1	PRSS1
MOL011604	Syringetin	Prostaglandin G/H synthase 2	PTGS2
MOL011604	Syringetin	Sodium channel protein type 5 subunit	SCN5A
MOL011604	Syringetin	DNA topoisomerase II	TOP2
