# Supplementary material for: Network Pharmacology-Based Strategy to Investigate the Pharmacological Mechanisms of Ginkgo biloba Extract for Aging
Source: Evid Based Complement Alternat Med. 2020 Jul 27;2020:8508491. doi: 10.1155/2020/8508491 (PMC7403930; doi:10.1155/2020/8508491)
Supplement: Supplementary Materials — Additional file 1: chemical information of main compounds in EGb. Additional file 2: targets of active ingredients. Additional file 3: target of aging. Additional file 4: potential targets of EGb for antiaging. [file 8508491.f1.zip › Additional file/Additional file 3.pdf]

Additional file 3 target of aging

Symbol	Name
GHR	growth hormone receptor
GHRH	growth hormone releasing hormone
SHC1	SHC (Src homology 2 domain containing) transforming protein 1
POU1F1	POU class 1 homeobox 1
PROP1	PROP paired-like homeobox 1
TP53	tumor protein p53
TERC	telomerase RNA component
TERT	telomerase reverse transcriptase
ATM	ATM serine/threonine kinase
PLAU	plasminogen activator, urokinase
ERCC2	excision repair cross-complementation group 2
ERCC8	excision repair cross-complementation group 8
WRN	Werner syndrome, RecQ helicase-like
LMNA	lamin A/C
IGF1R	insulin-like growth factor 1 receptor
TXN	thioredoxin
KL	klotho
E2F1	E2F transcription factor 1
PTPN11	protein tyrosine phosphatase, non-receptor type 11
NFKB2	nuclear factor of kappa light polypeptide gene enhancer in B-cells 2 (p49/p100)
STAT5B	signal transducer and activator of transcription 5B
STAT3	signal transducer and activator of transcription 3 (acute-phase response factor)
STAT5A	signal transducer and activator of transcription 5A
NRG1	neuregulin 1
DPP4	Dipeptidyl peptidase 4
HDAC3	histone deacetylase 3
GH1	growth hormone 1
IL7R	interleukin 7 receptor
IGF1	insulin-like growth factor 1 (somatomedin C)
IGF2	insulin-like growth factor 2
INS	insulin
NGF	nerve growth factor (beta polypeptide)
IRS1	insulin receptor substrate 1
PTPN1	protein tyrosine phosphatase, non-receptor type 1
IRS2	insulin receptor substrate 2
AKT1	v-akt murine thymoma viral oncogene homolog 1
PIK3CB	phosphatidylinositol-4,5-bisphosphate 3-kinase, catalytic subunit beta
NGFR	nerve growth factor receptor
HRAS	Harvey rat sarcoma viral oncogene homolog
MYC	v-myc avian myelocytomatosis viral oncogene homolog
EGFR	epidermal growth factor receptor
ERBB2	erb-b2 receptor tyrosine kinase 2
INSR	insulin receptor
NCOR1	nuclear receptor corepressor 1
NBN	nibrin
JUND	jun D proto-oncogene

IL2	interleukin 2
PDGFB	platelet-derived growth factor beta polypeptide
EGF	epidermal growth factor
IL2RG	interleukin 2 receptor, gamma
FOS	FBJ murine osteosarcoma viral oncogene homolog
PDGFRB	platelet-derived growth factor receptor, beta polypeptide
EPOR	Erythropoietin receptor
SST	somatostatin
PRKCD	protein kinase C, delta
PPARA	peroxisome proliferator-activated receptor alpha
RET	ret proto-oncogene
PLCG2	phospholipase C, gamma 2 (phosphatidylinositol-specific)
PEX5	peroxisomal biogenesis factor 5
TCF3	transcription factor 3
PARP1	poly (ADP-ribose) polymerase 1
BRCA1	breast cancer 1, early onset
PIN1	peptidylprolyl cis/trans isomerase, NIMA-interacting 1
PTEN	phosphatase and tensin homolog
CREBBP	CREB binding protein
HIF1A	hypoxia inducible factor 1, alpha subunit (basic helix-loop-helix transcription factor)
UBB	ubiquitin B
RPA1	replication protein A1, 70kDa
BLM	Bloom syndrome, RecQ helicase-like
BCL2	B-cell CLL/lymphoma 2
S100B	S100 calcium binding protein B
VCP	valosin containing protein
POLG	polymerase (DNA directed), gamma
IGFBP3	insulin-like growth factor binding protein 3
HSP90AA1	heat shock protein 90kDa alpha (cytosolic), class A
NR3C1	nuclear receptor subfamily 3, group C, member 1 (glucocorticoid receptor)
EGR1	early growth response 1
VEGFA	vascular endothelial growth factor A
ABL1	ABL proto-oncogene 1, non-receptor tyrosine kinase
BRCA2	breast cancer 2, early onset
TOP2A	topoisomerase (DNA) II alpha
TOP2B	topoisomerase (DNA) II beta
NFKB1	nuclear factor of kappa light polypeptide gene enhancer in B-cells 1
TOP1	topoisomerase (DNA) I
RAD51	RAD51 recombinase
UBE2I	ubiquitin-conjugating enzyme E2I
TNF	tumor necrosis factor
PDPK1	3-phosphoinositide dependent protein kinase 1
CEBPA	CCAAT/enhancer binding protein (C/EBP), alpha
CEBPB	CCAAT/enhancer binding protein (C/EBP), beta
MXI1	MAX interactor 1, dimerization protein
TGFB1	transforming growth factor, beta 1
ERCC6	excision repair cross-complementation group 6
STK11	serine/threonine kinase 11
EP300	E1A binding protein p300

APTX	aprataxin
PML	promyelocytic leukemia
GSK3B	glycogen synthase kinase 3 beta
HTT	huntingtin
PRKCA	protein kinase C, alpha
SSTR3	somatostatin receptor 3
HELLS	helicase, lymphoid-specific
APOC3	apolipoprotein C-III
EEF2	eukaryotic translation elongation factor 2
ERCC3	excision repair cross-complementation group 3
TERF1	telomeric repeat binding factor (NIMA-interacting) 1
PRKDC	protein kinase, DNA-activated, catalytic polypeptide
CAT	catalase
ERCC5	excision repair cross-complementation group 5
AR	androgen receptor
GTF2H2	general transcription factor IIH, polypeptide 2, 44kDa
XRCC5	X-ray repair complementing defective repair in Chinese hamster cells 5 (double-strand-break rejoining)
PCNA	proliferating cell nuclear antigen
FEN1	flap structure-specific endonuclease 1
FAS	Fas cell surface death receptor
TERF2	telomeric repeat binding factor 2
XRCC6	X-ray repair complementing defective repair in Chinese hamster cells 6
POLD1	polymerase (DNA directed), delta 1, catalytic subunit
BAX	BCL2-associated X protein
RB1	retinoblastoma 1
EMD	emerin
GRB2	growth factor receptor-bound protein 2
FOXO3	forkhead box O3
FOXO1	forkhead box O1
HSF1	heat shock transcription factor 1
XPA	xeroderma pigmentosum, complementation group A
MSRA	methionine sulfoxide reductase A
RECQL4	RecQ helicase-like 4
SOD2	superoxide dismutase 2, mitochondrial
SOD1	superoxide dismutase 1, soluble
FOXM1	forkhead box M1
COQ7	coenzyme Q7 homolog, ubiquinone (yeast)
CACNA1A	calcium channel, voltage-dependent, P/Q type, alpha 1A subunit
LRP2	low density lipoprotein receptor-related protein 2
AIFM1	apoptosis-inducing factor, mitochondrion-associated, 1
UCHL1	ubiquitin carboxyl-terminal esterase L1 (ubiquitin thiolesterase)
APP	amyloid beta (A4) precursor protein
APOE	apolipoprotein E
A2M	alpha-2-macroglobulin
SNCG	synuclein, gamma (breast cancer-specific protein 1)
PRDX1	peroxiredoxin 1
PON1	paraoxonase 1
RELA	v-rel avian reticuloendotheliosis viral oncogene homolog

IL6	interleukin 6
RGN	regucalcin
ATP5O	ATP synthase, H+ transporting, mitochondrial F1 complex, O subunit
RAD52	RAD52 homolog, DNA repair protein
TOP3B	topoisomerase (DNA) III beta
ERCC1	excision repair cross-complementation group 1
SIRT1	sirtuin 1
HDAC1	histone deacetylase 1
HSPA9	heat shock 70kDa protein 9 (mortalin)
GPX1	glutathione peroxidase 1
GSR	glutathione reductase
GSS	glutathione synthetase
GSTA4	glutathione S-transferase alpha 4
GSTP1	glutathione S-transferase pi 1
MT-CO1	mitochondrially encoded cytochrome c oxidase I
HSPD1	heat shock 60kDa protein 1 (chaperonin)
HSPA1A	heat shock 70kDa protein 1A
HSPA1B	heat shock 70kDa protein 1B
PCMT1	protein-L-isoaspartate (D-aspartate) O-methyltransferase
MAPK8	mitogen-activated protein kinase 8
YWHAZ	tyrosine 3-monooxygenase/tryptophan 5-monooxygenase activation protein, zeta
PTK2B	protein tyrosine kinase 2 beta
PTK2	protein tyrosine kinase 2
IL7	interleukin 7
MAPK14	mitogen-activated protein kinase 14
FGFR1	fibroblast growth factor receptor 1
SP1	Sp1 transcription factor
FLT1	fms-related tyrosine kinase 1
JUN	jun proto-oncogene
MED1	mediator complex subunit 1
MAPK9	mitogen-activated protein kinase 9
MAPK3	mitogen-activated protein kinase 3
HMGB1	high mobility group box 1
CCNA2	cyclin A2
HMGB2	high mobility group box 2
MAP3K5	mitogen-activated protein kinase kinase kinase 5
TAF1	TAF1 RNA polymerase II, TATA box binding protein (TBP)-associated factor
LMNB1	lamin B1
SDHC	succinate dehydrogenase complex, subunit C, integral membrane protein
FOXO4	forkhead box O4
HESX1	HESX homeobox 1
PIK3R1	phosphoinositide-3-kinase, regulatory subunit 1 (alpha)
BSCL2	Berardinelli-Seip congenital lipodystrophy 2 (seipin)
AGPAT2	1-acylglycerol-3-phosphate O-acyltransferase 2
BMI1	BMI1 proto-oncogene, polycomb ring finger
EEF1A1	eukaryotic translation elongation factor 1 alpha 1
TFAP2A	transcription factor AP-2 alpha (activating enhancer binding protein 2 alpha)

BDNF	brain-derived neurotrophic factor
CREB1	cAMP responsive element binding protein 1
ATF2	activating transcription factor 2
TBP	TATA box binding protein
APEX1	APEX nuclease (multifunctional DNA repair enzyme) 1
HBP1	HMG-box transcription factor 1
BUB1B	BUB1 mitotic checkpoint serine/threonine kinase B
PTGS2	prostaglandin-endoperoxide synthase 2 (prostaglandin G/H synthase and cyclooxygenase)
HSPA8	heat shock 70kDa protein 8
SIN3A	SIN3 transcription regulator family member A
CDK1	cyclin-dependent kinase 1
TFDP1	transcription factor Dp-1
DDIT3	DNA-damage-inducible transcript 3
POLA1	polymerase (DNA directed), alpha 1, catalytic subunit
MAPT	microtubule-associated protein tau
CTGF	connective tissue growth factor
HDAC2	histone deacetylase 2
MAX	MYC associated factor X
MXD1	MAX dimerization protein 1
MDM2	MDM2 proto-oncogene, E3 ubiquitin protein ligase
SUMO1	small ubiquitin-like modifier 1
H2AFX	H2A histone family, member X
HOXB7	homeobox B7
HOXC4	homeobox C4
JAK2	Janus kinase 2
ESR1	estrogen receptor 1
LEP	leptin
LEPR	leptin receptor
NFKBIA	nuclear factor of kappa light polypeptide gene enhancer in B-cells inhibitor, alpha
CLU	clusterin
MTOR	mechanistic target of rapamycin (serine/threonine kinase)
GHRHR	growth hormone releasing hormone receptor
CTNNB1	catenin (cadherin-associated protein), beta 1, 88kDa
PSEN1	presenilin 1
DLL3	delta-like 3 (Drosophila)
CDKN2A	cyclin-dependent kinase inhibitor 2A
PPP1CA	protein phosphatase 1, catalytic subunit, alpha isozyme
DBN1	drebrin 1
NOG	noggin
ELN	elastin
ATR	ATR serine/threonine kinase
UCP3	uncoupling protein 3 (mitochondrial, proton carrier)
ZMPSTE24	zinc metallopeptidase STE24
TP63	tumor protein p63
UCP2	uncoupling protein 2 (mitochondrial, proton carrier)
POLB	polymerase (DNA directed), beta
GCLC	glutamate-cysteine ligase, catalytic subunit
GCLM	glutamate-cysteine ligase, modifier subunit
SIRT6	sirtuin 6
BUB3	BUB3 mitotic checkpoint protein

RAE1	ribonucleic acid export 1
PMCH	pro-melanin-concentrating hormone
MLH1	mutL homolog 1
CSNK1E	casein kinase 1, epsilon
STUB1	STIP1 homology and U-box containing protein 1, E3 ubiquitin protein ligase
PPM1D	protein phosphatase, Mg2+/Mn2+ dependent, 1D
CHEK2	checkpoint kinase 2
PCK1	phosphoenolpyruvate carboxykinase 1 (soluble)
ARHGAP1	Rho GTPase activating protein 1
CDC42	cell division cycle 42
ARNTL	aryl hydrocarbon receptor nuclear translocator-like
CLOCK	clock circadian regulator
HIC1	hypermethylated in cancer 1
PAPPA	pregnancy-associated plasma protein A, pappalysin 1
ADCY5	adenylate cyclase 5
PPARGC1A	peroxisome proliferator-activated receptor gamma, coactivator 1 alpha
GPX4	glutathione peroxidase 4
UCP1	uncoupling protein 1 (mitochondrial, proton carrier)
FGF23	fibroblast growth factor 23
EFEMP1	EGF containing fibulin-like extracellular matrix protein 1
ERCC4	excision repair cross-complementation group 4
CETP	cholesteryl ester transfer protein, plasma
PPARG	peroxisome proliferator-activated receptor gamma
AGTR1	angiotensin II receptor, type 1
CISD2	CDGSH iron sulfur domain 2
EEF1E1	eukaryotic translation elongation factor 1 epsilon 1
EPS8	epidermal growth factor receptor pathway substrate 8
KCNA3	potassium channel, voltage gated shaker related subfamily A, member 3
SIRT7	sirtuin 7
SLC13A1	solute carrier family 13 (sodium/sulfate symporter),
SOCS2	suppressor of cytokine signaling 2
TPP2	tripeptidyl peptidase II
TP53BP1	tumor protein p53 binding protein 1
SIRT3	sirtuin 3
NCOR2	nuclear receptor corepressor 2
SUN1	Sad1 and UNC84 domain containing 1
BAK1	BCL2-antagonist/killer 1
IGFBP2	insulin-like growth factor binding protein 2, 36kDa
PYCR1	pyrroline-5-carboxylate reductase 1
TP73	tumor protein p73
CNR1	cannabinoid receptor 1 (brain)
NFE2L2	nuclear factor, erythroid 2-like 2
CDKN1A	cyclin-dependent kinase inhibitor 1A (p21, Cip1)
PDGFRA	platelet-derived growth factor receptor, alpha polypeptide
PIK3CA	phosphatidylinositol-4,5-bisphosphate 3-kinase, catalytic subunit alpha
C1QA	complement component 1, q subcomponent, A chain
CDKN2B	cyclin-dependent kinase inhibitor 2B (p15, inhibits
EIF5A2	eukaryotic translation initiation factor 5A2

MIF	macrophage migration inhibitory factor (glycosylation-inhibiting factor)
DGAT1	diacylglycerol O-acyltransferase 1
MT1E	metallothionein 1E
FGF21	fibroblast growth factor 21
HTRA2	HtrA serine peptidase 2
GSK3A	glycogen synthase kinase 3 alpha
NUDT1	nudix (nucleoside diphosphate linked moiety X)-type
IKBKB	inhibitor of kappa light polypeptide gene enhancer in B-cells, kinase beta
SQSTM1	sequestosome 1
CDK7	cyclin-dependent kinase 7
GRN	granulin
SERPINE1	serpin peptidase inhibitor, clade E (nexin, plasminogen activator inhibitor type 1), member 1
SPRTN	SprT-like N-terminal domain
RICTOR	RPTOR independent companion of MTOR, complex 2
CTF1	cardiotrophin 1
TRAP1	TNF receptor-associated protein 1
TRPV1	transient receptor potential cation channel subfamily V member 1
NFE2L1	nuclear factor, erythroid 2-like 1
IFNB1	Interferon beta
GDF11	growth differentiation factor 11
