# Supplementary material for: Network Pharmacology-Based Strategy to Investigate the Pharmacological Mechanisms of Ginkgo biloba Extract for Aging
Source: Evid Based Complement Alternat Med. 2020 Jul 27;2020:8508491. doi: 10.1155/2020/8508491 (PMC7403930; doi:10.1155/2020/8508491)
Supplement: Supplementary Materials — Additional file 1: chemical information of main compounds in EGb. Additional file 2: targets of active ingredients. Additional file 3: target of aging. Additional file 4: potential targets of EGb for antiaging. [file 8508491.f1.zip › Additional file/Additional file 4.pdf]

Additional file 4 Potential targets of EGb for anti-aging

UniProt	Gene name	Target Name
P05067	APP	Amyloid-beta precursor protein
P10276	AR	Androgen receptor
P04637	TP53	Cellular tumor antigen p53
P37231	PPARG	Peroxisome proliferator-activated receptor gamma
P06612	TOP1	DNA topoisomerase 1
P00533	EGFR	Epidermal growth factor receptor
P03372	ESR1	Estrogen receptor
P09211	GSTP1	Glutathione S-transferase P
P49841	GSK3B	Glycogen synthase kinase-3 beta
P06213	INSR	Insulin receptor
P60568	IL2	Interleukin-2
P05231	IL6	Interleukin-6
Q16539	MAPK14	Mitogen-activated protein kinase 14
P20248	CCNA2	Cyclin-A2
P06400	RB1	Retinoblastoma-associated protein
P05412	JUN	Transcription factor AP-1
P01375	TNF	Tumor necrosis factor
P35968	KDR	Vascular endothelial growth factor receptor 2
P10415	BCL2	Bcl-2-related protein A1
P27169	PON1	Serum paraoxonase/arylesterase 1
P00441	SOD1	Superoxide dismutase
P00749	PLAU	Urokinase-type plasminogen activator
P04150	NR3C1	Glucocorticoid receptor
P45983	MAPK8	Mitogen-activated protein kinase 8
P17252	PRKCA	Protein kinase C alpha type
Q05655	PRKCD	Protein kinase C delta type
P35354	PTGS2	Prostaglandin G/H synthase 2
P27487	DPP4	Dipeptidyl peptidase 4
